# Mitochondrial Ubiquitin Ligase MARCH5 Promotes TLR7 Signaling by Attenuating TANK Action

**DOI:** 10.1371/journal.ppat.1002057

**Published:** 2011-05-19

**Authors:** He-Xin Shi, Xing Liu, Qiang Wang, Pei-Pei Tang, Xin-Yi Liu, Yu-Fei Shan, Chen Wang

**Affiliations:** Laboratory of Molecular Cell Biology, Institute of Biochemistry and Cell Biology, Shanghai Institutes for Biological Sciences, Chinese Academy of Sciences, Shanghai, China; North Carolina State University, United States of America

## Abstract

The signaling of Toll-like receptors (TLRs) is the host's first line of defense against microbial invasion. The mitochondrion is emerging as a critical platform for antiviral signal transduction. The regulatory role of mitochondria for TLR signaling remains to be explored. Here, we show that the mitochondrial outer-membrane protein MARCH5 positively regulates TLR7 signaling. Ectopic expression or knockdown of MARCH5 enhances or impairs NF-κB-mediated gene expression, respectively. MARCH5 interacts specifically with TANK, and this interaction is enhanced by R837 stimulation. MARCH5 catalyzes the K63-linked poly-ubiquitination of TANK on its Lysines 229, 233, 280, 302 and 306, thus impairing the ability of TANK to inhibit TRAF6. Mislocalization of MARCH5 abolishes its action on TANK, revealing the critical role of mitochondria in modulating innate immunity. Arguably, this represents the first study linking mitochondria to TLR signaling.

## Author Summary

In 2005, MAVS was characterized as the critical adaptor protein for the signal transduction of RIG-I-like receptors (RLRs). This provided the first link between mitochondria and the intracellular antiviral defense system. From then on, exploring the potential functions of novel mitochondrial proteins in microbe-host interactions became a rapidly expanding frontier. Notably, it remains unknown whether mitochondrial proteins can directly regulate TLR signaling. Here, we demonstrate that the mitochondrial protein MARCH5 positively modulates TLR7 signaling. Our study reveals that MARCH5 is a novel E3 ubiquitin ligase and catalyzes the K63-linked poly-ubiquitination of TANK. This modification releases the inhibitory effects of TANK on TRAF6. Arguably, this represents the first study linking mitochondria to TLR signaling, shedding new light on the role of mitochondria in the proinflammatory response.

## Introduction

Germline-encoded pattern recognition receptors (PRRs) play pivotal roles in sensing a wide range of invading pathogens, via recognizing conserved microbial signature molecules (PAMPs, pathogen associated molecular patterns). As molecular switches that register microbial infection, these receptors promptly initiate innate immune responses and subsequently prime the adaptive immune system to eliminate the pathogens [1], [2], [3]. Toll-like receptors (TLRs) are one major class of PRRs. To date, 13 members of the TLR family have been identified in mammals, of which TLR4 recognizes lipopolysaccharide (LPS) from gram-negative bacteria, and TLR7 senses viral single strand RNA (ssRNA) [2]. Both TLR4 and TLR7 can induce the robust expression of proinflammatory cytokines and type I interferons (IFNs).

Recent years have witnessed an incredible gain in knowledge about the TLR signaling cascades. For example, in response to stimuli, TLR7 triggers the recruitment of MyD88 via its Toll-interleukin 1 receptor (IL-1R) homology (TIR) domain, which in turn recruits IRAK4 and IRAK1. IRAK4 then activates IRAK-1 by phosphorylation. As a result, the IRAKs dissociate from MyD88 and interact with TRAF6, a ubiquitin E3 ligase [4], [5], [6]. Together with Ubc13 and Uev1A, TRAF6 catalyzes the formation of lysine 63 (K63)-linked poly-ubiquitin chains, which serve as the anchoring platform for a protein complex that includes TRAF6, TRAF3, IKKα, and IRF7, leading ultimately to the induction of type I IFNs and ISGs (interferon-inducible genes) [7], [8]. In the meantime, TAK1 is recruited to the TRAF6 protein complex, resulting in the activation of NF-κB and the induction of proinflammatory cytokines [9].

Given the critical roles of TLR signaling in innate immunity, multiple layers of stringent regulations are employed to ensure that the strength and duration of the TLR signal is appropriate for any given immune response. Several proteins have been demonstrated to modulate the TLR signaling pathways, such as A20, CYLD, IRAKM, ST2, SIGIRR and SOCS1 [10], [11], [12], [13], [14], [15], [16]. Importantly, TANK (also called I-TRAF) was initially characterized as a TRAF binding protein [17], [18]. Recently, the *in vivo* function of TANK has been further clarified. Surprisingly, TANK is not essential for interferon induction and instead is a potent negative regulator for TLR-mediated induction of proinflammatory cytokines [19]. How TANK specifically modulates NF-κB signaling upon TLR activation remains to be determined.

Mitochondria are rapidly emerging as important platforms for intracellular antiviral signaling. MAVS (also known as IPS-1/VISA/Cardif) is the first mitochondrial protein identified as a critical component of the RIG-I/MDA5 signaling pathway [20]. Following this, several more mitochondrial proteins have been implicated in modulating this same antiviral signaling pathway, such as NLRX1, STING (also known as MITA) and MFN1 [21], [22], [23], [24]. We have recently discovered that the mitochondrial outer-membrane receptor TOM70 mediates IRF3 activation downstream of MAVS [25], [26], [27].

Notably, whether any mitochondrial protein(s) can regulate TLR signaling remains an open question. It was recently reported that two ubiquitin E3 ligases constitutively express on mitochondria, MARCH5/MITOL/RNF153 and GIDE (Growth Inhibition and Death E3 Ligase). MARCH family proteins are characterized by harboring a RING-CH domain and multiple trans-membrane domains. Interestingly, many RING proteins function as E3 ubiquitin ligases. Some of the MARCH proteins (MARCH1, MARCH8) appear to either directly or indirectly modulate immune functions by controlling the surface turnover of immune regulatory molecules on the plasma membrane [28]. MARCH5 (also named MARCH-V/MITOL/RNF153) was recently identified as a new member in the MARCH family [29], [30]. Preliminary characterization uncovered MARCH5 as a novel mitochondrial protein. Until recently, little was known about the potential function of MARCH5. Several recent studies have determined that MARCH5 can potentially modulate mitochondrial fission and the morphology of mitochondria [29], [30], [31].

In this study, we reveal that the mitochondrial protein MARCH5 is a novel E3 ubiquitin ligase and a positive regulator for TLR7 signaling. MARCH5 catalyzes the K63-linked poly-ubiquitination of TANK on its Lysines 229, 233, 280, 302 and 306. This modification releases the inhibitory effects of TANK toward TRAF6. Consequently, ectopic expression or knockdown of MARCH5 enhances or impairs NF-κB-mediated proinflammatory gene expression, respectively, in response to TLR7 activation. Interestingly, the localization of MARCH5 on mitochondria is indispensable for its regulatory action, demonstrating a new role for mitochondria in the proinflammatory response. To our knowledge, this is the first report that links mitochondria to TLR signaling.

## Results

### Identification of MARCH5 as a new regulator of TLR signaling

**Figure 1:**
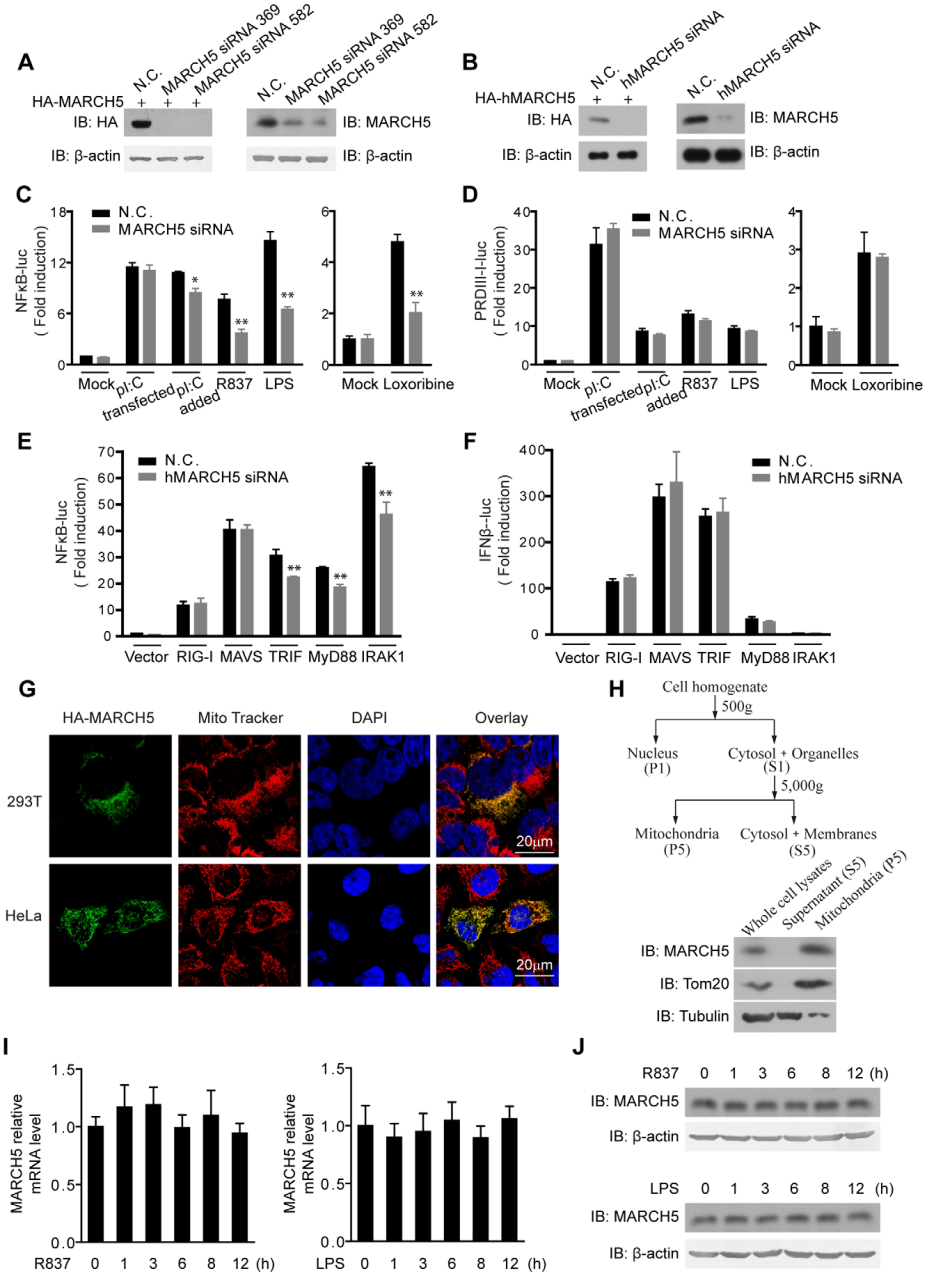
Identification of MARCH5 in TLR7 signaling pathway. A, HEK293T cells were transfected with HA-MARCH5 and then treated with the nonspecific control (N.C.) or MARCH5 siRNA (left panel). Raw264.7 cells were transfected with the negative control (N.C.) or MARCH5 siRNA (right panel). Cell lysates were immunoblotted with the indicated antibodies. B, HEK293T cells were transfected with HA-hMARCH5 and then treated with the nonspecific control (N.C.) or hMARCH5 siRNA (left panel). HEK293T cells were transfected with the negative control (N.C.) or hMARCH5 siRNA (right panel). Cell lysates were immunoblotted with the indicated antibodies. C and D, the nonspecific control (N.C.) or MARCH5 siRNA were transfected into Raw264.7 cells with NF-κB (C) or PRDIII-I reporter plasmids (D), respectively. Forty-eight hours after transfection, cells were stimulated with poly(I:C) (2 µg/ml, transfected), poly(I:C) (50 µg/ml, added to the culture medium), R837 (10 µg/ml), Loxoribine (50 µM) or LPS (100 ng/ml) for 6 h before luciferase assays were performed. A pTK-Renilla reporter was used to normalize data. E and F, the nonspecific control (N.C.) or hMARCH5 siRNA were transfected into HEK293T cells together with NF-κB (E) or IFNβ reporter plasmids (F), respectively. Forty-eight hours after transfection, cells were transfected again with RIG-I, MAVS, TRIF, MyD88 or IRAK1 for 16 h before luciferase assays were performed. A pTK-Renilla reporter was used to normalize data. G, HA-MARCH5 was transfected into HEK293T cells (upper panel) or HeLa cells (lower panel), which were then stained with an anti-HA antibody and imaged by confocal microscopy. The mitochondria were stained with MitoTracker. H, HEK293T cells were fractionated as shown in diagram (top panel). The corresponding fractions were analyzed by immunoblotting with the indicated antibodies (bottom panel). I and J, Raw264.7 cells were stimulated with R837 (10 µg/ml) or LPS (100 ng/ml), respectively, for the indicated time periods. The mRNA level of MARCH5 was measured by quantitative PCR (I), and the protein level of MARCH5 was measured by immunoblotting with an anti-MARCH5 antibody (J). Data from C–F and I are presented as means ± S.D. from three independent experiments. *, *P*<0.05; **, *P*<0.01.

Poly-ubiquitination is a major mechanism for regulating innate immunity. Because the mitochondrial protein MAVS was recently reported to be modified by both K48- and K63- linked poly-ubiquitin [32], [33], [34], we speculated whether these post-translational modifications were mediated by any mitochondrial ubiquitin E3 ligase. We noticed that MARCH5/MITOL/RNF153 is constitutively expressed in mitochondria and contains a RING domain, a signature of ubiquitin E3 ligases. To explore the potential role of MARCH5, we screened effective siRNAs against MARCH5 (MARCH5 siRNA 369 and MARCH5 siRNA 582 for mouse, hMARCH5 siRNA for human) (Fig. 1A, B). It was found that knockdown of endogenous MARCH5 did not affect κB-luciferase and PRDIII-I-luciferase reporters (responsive to NF-κB and IRF3/7 activation, respectively) stimulated by Sendai virus or transfected poly(I:C). Consistently, silencing of MARCH5 produced no effect on RIG-I- or MAVS-mediated activation of κB-luciferase or IFN-β-luciferase reporters (Fig. 1 E, F). Nor did MARCH5 produce any effect on MAVS poly-ubiquitination (Fig. S7). These indicate that MARCH5 is dispensable for RIG-I/MDA5 signaling.

Surprisingly, activation of κB-luciferase reporter was apparently impaired upon MARCH5 depletion in response to TLR3, TLR7 or TLR4 signaling in Raw264.7 cells when stimulated with extracellular poly(I:C), imiquimod (R837), Loxoribine or LPS, respectively (Fig. 1C). Notably, the impairment was more significant for TLR7 and TLR4 than for TLR3. Interestingly, activation of the PRDIII-I-luciferase reporter was barely modulated by MARCH5 under these stimuli (Fig. 1D). Apparently, MARCH5 could modulate TRIF-, MyD88- or IRAK1-induced κB-luciferase activity (Fig. 1E). These data suggest that MARCH5 is possibly critical for NF-κB activation downstream of TLRs but is dispensable for IRF3/7 activation.

In addition, confocal microscopy and fractionation analysis confirmed that MARCH5 was predominantly expressed on mitochondria (Fig. 1G, H). R837 and LPS stimulation did not influence the expression of MARCH5 (Fig. 1I, J).

### MARCH5 potentiates TLR7-mediated NF-κB activation

**Figure 2:**
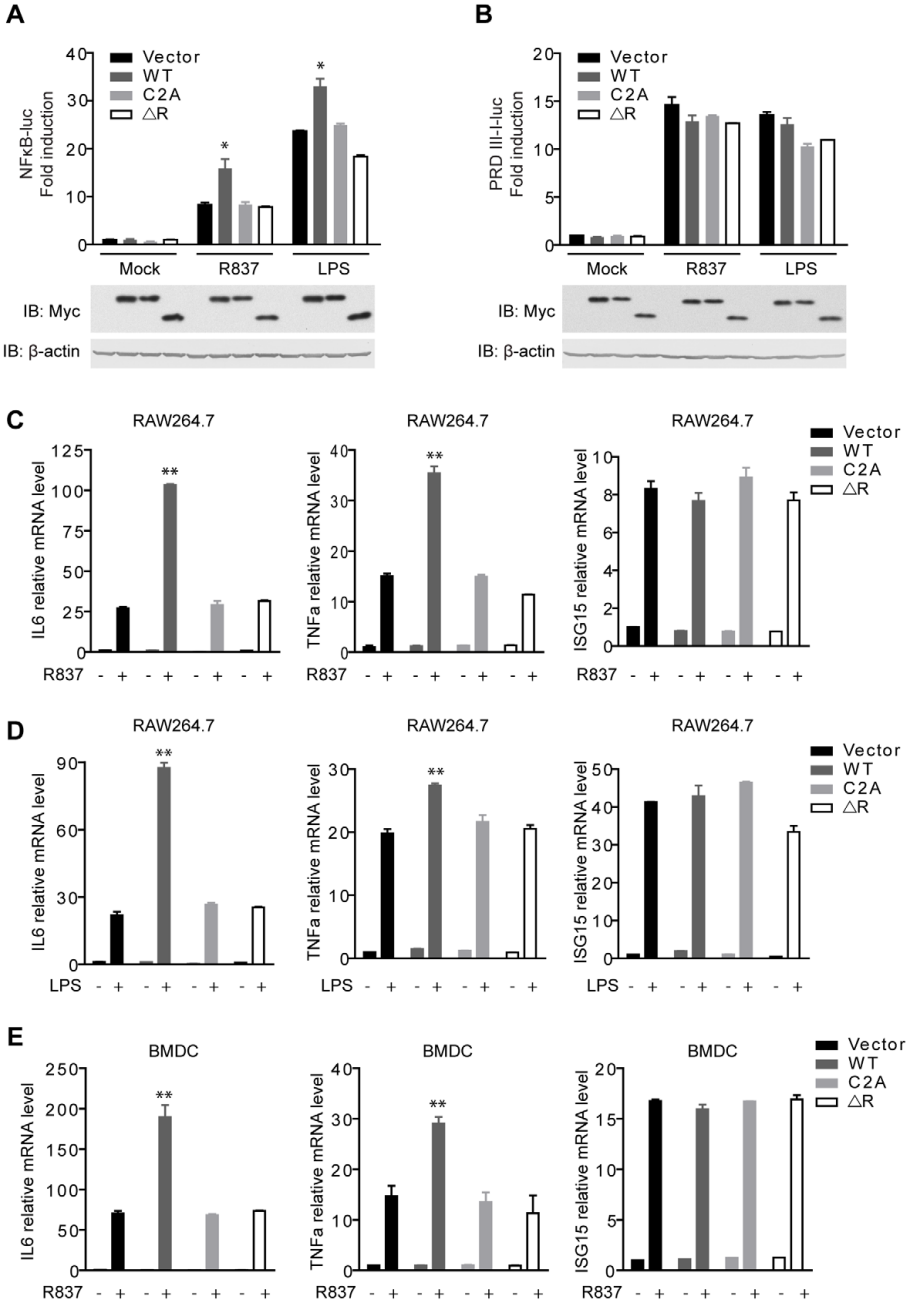
MARCH5 synergizes TLR7- induced NF-κB activation. A and B, Equal amounts of the indicated Myc-tagged MARCH5 constructs were transfected into Raw264.7 cells together with NF-κB (A) or PRDIII-I reporter plasmids (B). Twenty-four hours after transfection, cells were stimulated with R837 (10 µg/ml) or LPS (100 ng/ml) for 6 h before luciferase assays were performed. C and D, induction of IL6, TNFα, and ISG15 mRNA by R837 (C) or LPS (D) stimulation in the presence of the indicated plasmids in RAW264.7 cells were measured by quantitative PCR. E, BMDCs (bone marrow-derived dendritic cells) were transfected with the indicated plasmids and then stimulated by R837 (10 µg/ml). Induction of IL6, TNFα, and ISG15 mRNAs was measured by qPCR. Data from A–E are presented as means ± S.D. from three independent experiments. *, *P*<0.05; **, *P*<0.01. WT, MARCH5 wild type; C2A, MARCH5 C65, 68A mutant; and ΔR, MARCH5 RING domain deletion mutant.

To substantiate these observations, the introduction of wild-type (WT) MARCH5 into Raw264.7 cells potentiated the activation of the κB-luciferase reporter upon R837 or LPS stimulation (Fig. 2A). In contrast, the activation of PRDIII-I-luciferase reporter was unaffected by ectopic expression of MARCH5 under the same stimuli (Fig. 2B).

Furthermore, we investigated whether MARCH5 modulated the expression of NF-κB-responsive genes (IL6 and TNFα) induced by R837 using the assays of qPCR (quantitative PCR) and ELISA (enzyme-linked immunosorbent assay). As shown in Fig. 2C and Fig. S1A, MARCH5 displayed synergic effects on the induction of NF-κB-responsive genes. Similarly, MARCH5 synergized NF-κB activation stimulated by LPS (Fig. 2D and Fig. S1B). However, this did not apply to the induction of ISG15 mRNA, which is regulated by IRF3/7 (Fig. 2C, D).

To make the experiments more physiologically relevant, we examined whether MARCH5 regulated NF-κB-induced gene expression in primary cells. Consistently, ectopic expression of MARCH5 markedly potentiated the expression of endogenous NF-κB-responsive genes (IL6 and TNFα) induced by R837 in BMDCs (bone marrow-derived dendritic cells) (Fig. 2E).

To explore the potential role of MARCH5 RING domain, we generated MARCH5 C2A (C65/68S) and MARCH5 ΔRING (Δ12-70 a.a.), both of which abrogated the potential E3 ubiquitin ligase activity of MARCH5 (Fig. S5A). As shown in Fig. 2A, exogenous expression of MARCH5 C2A and MARCH5 ΔRING failed to synergize the induction of NF-κB-responsive reporter upon R837 or LPS stimulation. Consistently, the expression of NF-κB-responsive genes (IL6 and TNFα) induced by R837 or LPS was barely affected when MARCH5 C2A or MARCH5 ΔRING was ectopically expressed (Fig. 2C, D, E and Fig. S1A, B). Apparently, ectopic expression of MARCH5 has no effect on the cell cycle or apoptosis (Fig. S2). Taken together, these data suggest that MARCH5 specifically potentiates TLR signaling, and this effect is dependent on its E3 ubiquitin ligase activity.

### Knockdown of MARCH5 attenuates TLR7-mediated NF-κB activation

**Figure 3:**
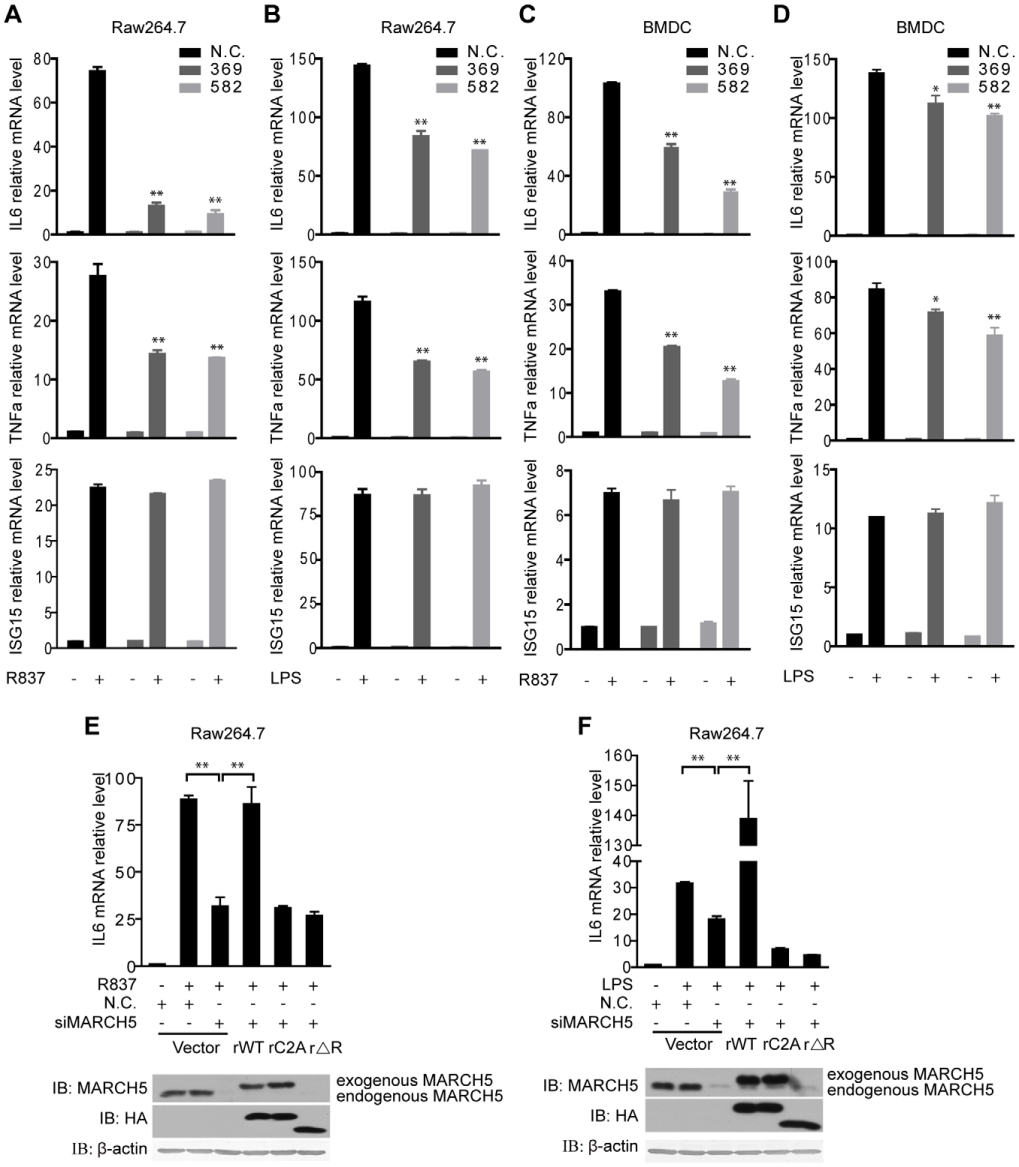
Knockdown of MARCH5 impairs TLR7- induced NF-κB activation. A and B, The indicated siRNAs were transfected into Raw264.7 cells. Induction of IL6, TNFα, and ISG15 mRNA was measured by quantitative PCR after R837 (10 µg/ml) (A) or LPS (100 ng/ml) (B) stimulation. C and D, The indicated siRNAs were transfected into BMDCs. Induction of IL6, TNFα, and ISG15 mRNA was measured by quantitative PCR after R837 (10 µg/ml) (C) or LPS (100 ng/ml) (D) stimulation. E and F, Raw264.7 cells were transfected with the negative control (N.C.) or MARCH5 siRNA and then rescued with the indicated siRNA-resistant MARCH5 constructs. After R837 (10 µg/ml) (E) or LPS (100 ng/ml) (F) stimulation, induction of IL6 mRNA was measured by quantitative PCR. Data in A–F are presented as means ± S.D. from three independent experiments. *, *P*<0.05; **, *P*<0.01.

Next, we explored the effect of MARCH5 knockdown on the expression of endogenous NF-κB-responsive genes, stimulated by R837 or LPS in Raw264.7 cells. As expected, MARCH5 knockdown attenuated the induction of NF-κB-responsive genes (IL6 and TNFα), but not that of an IRF3/7-responsive gene (ISG15) (Fig. 3A, B and Fig. S1C, D).

We further investigated the function of MARCH5 in primary cells. BMDCs were transfected with siRNAs against MARCH5, followed by R837 or LPS stimulation. Consistently, knockdown of MARCH5 markedly attenuated the expression of endogenous NF-κB-responsive genes in BMDCs (Fig. 3C, D).

To rule out potential off-target effects of the MARCH5 siRNA, we generated several RNA interference (RNAi)-resistant HA-MARCH5 constructs, namely rMARCH5 WT, rMARCH5 C2A and rMARCH5 ΔRING, in which silent mutations were introduced into the sequence targeted by the siRNA without changing the amino acid sequence of the corresponding proteins. Raw264.7 cells were first transfected with control or MARCH5 siRNA followed by transfection of control or indicated rMARCH5 plasmids, respectively. Then the induction of IL6 mRNA was measured after R837 or LPS stimulation. As shown in Fig. 3E and Fig. 3F, the induction of IL6 was restored by rMARCH5 WT, but not rescued by rMARCH5 C2A and rMARCH5 ΔRING. Apparently, knockdown of MARCH5 influences neither the cell cycle nor apoptosis (Fig. S3). Collectively, these results indicate that MARCH5 is a positive regulator of TLR-mediated NF-κB activation.

### MARCH5 specifically interacts with TANK

**Figure 4:**
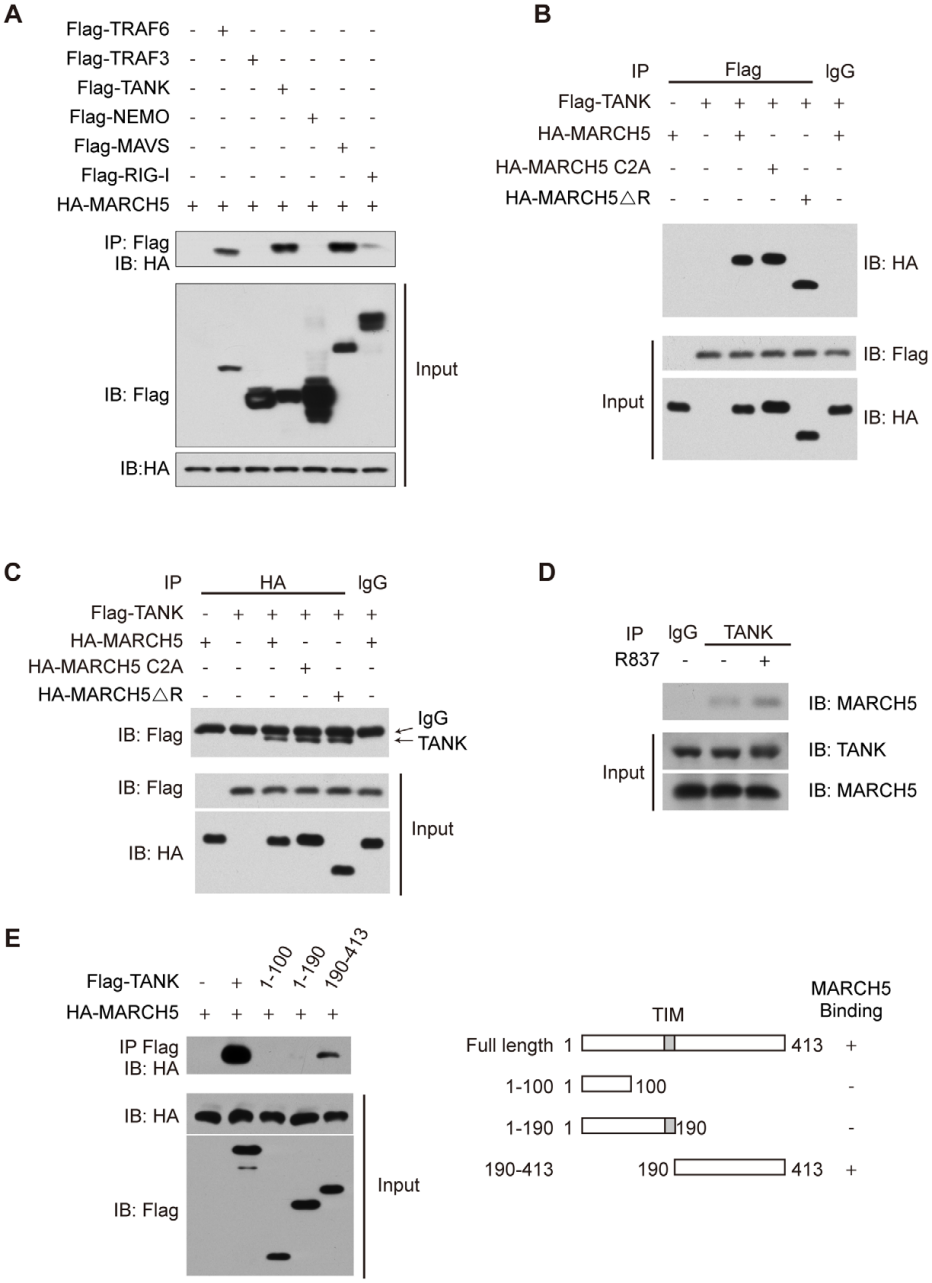
MARCH5 interacts with TANK. A, The indicated Flag tagged protein constructs were individually transfected into HEK293T cells along with HA-MARCH5. Then, equal amounts of cell lysates were immunoprecipitated with an anti-Flag antibody. The immunoprecipitates were immunoblotted with an anti-HA antibody. B, HEK293T cells were cotransfected with the indicated constructs. Then, equal amounts of cell lysates were immunoprecipitated (IP) with anti-IgG or anti-Flag antibodies. The immunoprecipitates were immunoblotted with an anti-HA antibody. C, HEK293T cells were cotransfected with the indicated constructs. Then, equal amounts of cell lysates were immunoprecipitated with anti-IgG or anti-HA antibodies. The immunoprecipitates were immunoblotted with an anti-Flag antibody. D, After mock or R837 (10 µg/ml) stimulation, lysates from Raw264.7 cells were immunoprecipitated with anti-TANK antibody or anti-IgG and then immunoblotted with an anti-MARCH5 antibody. E, Schematic diagram of TANK and its truncation mutants (right) (TIM, TRAF-interaction motif). Flag-TANK mutants were individually transfected into HEK293T cells along with HA-MARCH5. The cell lysates were immunoprecipitated with an anti-Flag antibody and then immunoblotted with the indicated antibodies (left).

To address the mechanism of MARCH5 action, we explored whether MARCH5 could interact with TLR signaling proteins. A co-immunoprecipitation assay revealed that MARCH5 interacted with TRAF6, TANK and MAVS but did not interact with TRAF3, NEMO or RIG-I (Fig. 4A). Interestingly, MARCH5 did not catalyze the ubiquitination of any of the indicated proteins except TANK (Fig. S7). Therefore, we further probed the interaction between MARCH5 and TANK.

As shown in Fig. 4B, HA-MARCH5 co-immunoprecipitated with Flag-TANK, but not with control IgG. Similarly, Flag-TANK co-immunoprecipitated with HA-MARCH5, but not with control IgG (Fig. 4C). Notably, HA-MARCH5 C2A and HA-MARCH5 ΔRING could interact with Flag-TANK as well (Fig. 4B, C). We subsequently confirmed the endogenous interaction between MARCH5 and TANK (Fig. 4D). Interestingly, the endogenous interaction between MARCH5 and TANK was enhanced by R837 stimulation (Fig. 4D).

A series of Flag-TANK deletion mutants were generated and individually transfected into HEK293T cells along with HA-MARCH5. It was observed that the C terminal region of TANK (amino acids 190 to 413) mediated this interaction (Fig. 4E).

We went on to investigate the sub-cellular localization of endogenous TANK. Confocal microscopy revealed that TANK displayed a punctate staining pattern in the cytoplasm of resting cells. Interestingly, TANK partially co-localized with mitochondria upon R837 or LPS stimulation, suggesting that TANK was dynamically recruited to mitochondria in response to these stimuli (Fig. S4). Collectively, these results indicate that MARCH5 is a new TANK binding protein *in vivo*.

### MARCH5 catalyzes K63-linked poly-ubiquitination of TANK

A couple of recent studies implicated MARCH5 as an E3 ubiquitin ligase that catalyzes the ubiquitination of hFis1 and Drp1, causing their degradation [31]. *In vitro* ubiquitination assays confirmed that the MARCH5 RING domain can catalyze the formation of both K48- and K63-linked polyUb chains, whereas MARCH5-C2A or MARCH5 ΔRING cannot (Fig. S5).

**Figure 5:**
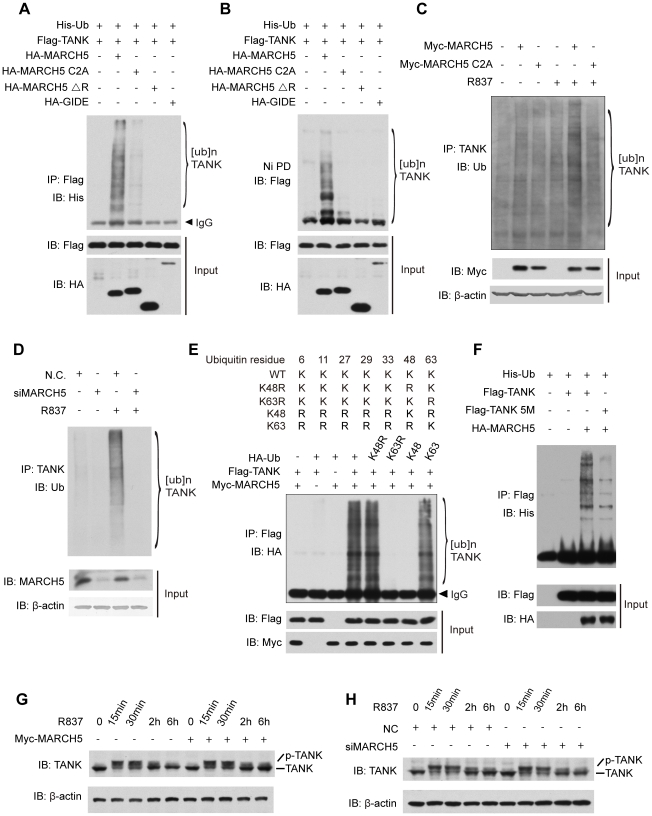
MARCH5 catalyzes K63-linked poly-ubiquitination of TANK. A and B, HEK293T cells were transfected with the indicated plasmids. Twenty-four hours after transfection, cell lysates were subjected to immunoprecipitation (A) or Ni-NTA pulldown (Ni PD) (B) and then immunoblotted with the indicated antibodies. C, Raw264.7 cells were transfected with the indicated plasmids and then stimulated with R837 (10 µg/ml). Cell lysates were subjected to immunoprecipitation and then immunoblotted with the indicated antibodies. D, Raw264.7 cells were transfected with the indicated siRNAs. After R837 stimulation, cell lysates were subjected to immunoprecipitation and immunoblotted with the indicated antibodies. E, HEK293T cells expressing the indicated plasmids were subjected to immunoprecipitation and then immunoblotted with the indicated antibodies. F, HEK293T cells were transfected with the indicated plasmids. Twenty-four hours after transfection, cell lysates were subjected to immunoprecipitation and then immunoblotted with the indicated antibodies. G and H, Raw264.7 cells transfected with the indicated plasmids (G) or siRNAs (H) were stimulated with R837 for the indicated time periods. The cell lysates were immunoblotted with an anti-TANK antibody. 5M, K229, 233, 280, 302 and 306R.

Our above data revealed the importance of this E3 ubiquitin ligase activity for regulating TLR signaling (Fig. 2). Therefore, we wondered whether TANK was a new ubiquitination target of MARCH5. In HEK293T cells, Flag-TANK and His-Ubiquitin was co-transfected with MARCH5 (WT), MARCH5 C2A, MARCH5 ΔRING or GIDE, respectively. The cell lysates were subjected to immunoprecipitation of Flag-TANK (Fig. 5A) or Ni-NTA pulldown of His-Ubiquitin (Fig. 5B). Then the precipitates were probed with indicated antibodies as seen in Fig. 5A and Fig. 5B. Notably, TANK was markedly poly-ubiquitinated in the presence of MARCH5 (WT). In contrast, MARCH5 C2A, MARCH5 ΔRING and GIDE could not catalyze ubiquitination of TANK (Fig. 5A, B). In addition, ectopic-expression of MARCH5 enhanced poly-ubiquitination of TANK in Raw264.7 cells upon R837 stimulation, whereas MARCH5 C2A had no such effect (Fig. 5C). Consistently, knockdown of MARCH5 attenuated this poly-ubiquitination of endogenous TANK (Fig. 5D). These data indicate that MARCH5 is a novel E3 ubiquitin ligase for TANK.

It was recently reported that TANK was modified by K63-linked polyubiquitination via (an) unknown E3 ubiquitin ligase(s) [35]. We further tested whether MARCH5 could fulfill this function. A panel of ubiquitin mutants was employed including those containing a point mutation at lysine 48 (K48R) or 63 (K63R) or lacking all lysines except K48 (K48-only ubiquitin) or K63 (K63-only ubiquitin) (Fig. 5E upper panel). As expected, MARCH5 catalyzed TANK poly-ubiquitination in the presence of wild type ubiquitin. Importantly, TANK was poly-ubiquitinated as well when using the K48R or K63 ubiquitin whereas poly-ubiquitination of TANK disappeared when using the K63R or K48 ubiquitin (Fig. 5E lower panel). This firmly established that MARCH5 facilitated the synthesis of K63- rather than K48- linked poly-ubiquitin chains onto TANK.

To identify the potential ubiquitination sites on TANK, we carried out a systematic lysine (K) to arginine (R) mutation scanning. It was observed that ubiquitination of the TANK(5M) mutant was markedly impaired (Fig. 5F). Notably, the TANK(5M) mutant could interact with MARCH5 as well as wild-type TANK (Fig. S6), indicating that these five lysines, 229, 233, 280, 302 and 306, were the major ubiquitination sites on TANK. Consistently, ectopic expression or knockdown of MARCH5 apparently did not affect the stability of endogenous TANK (Fig. 5G, H).

### MARCH5 modulates TRAF6 auto-ubiquitination through TANK

**Figure 6:**
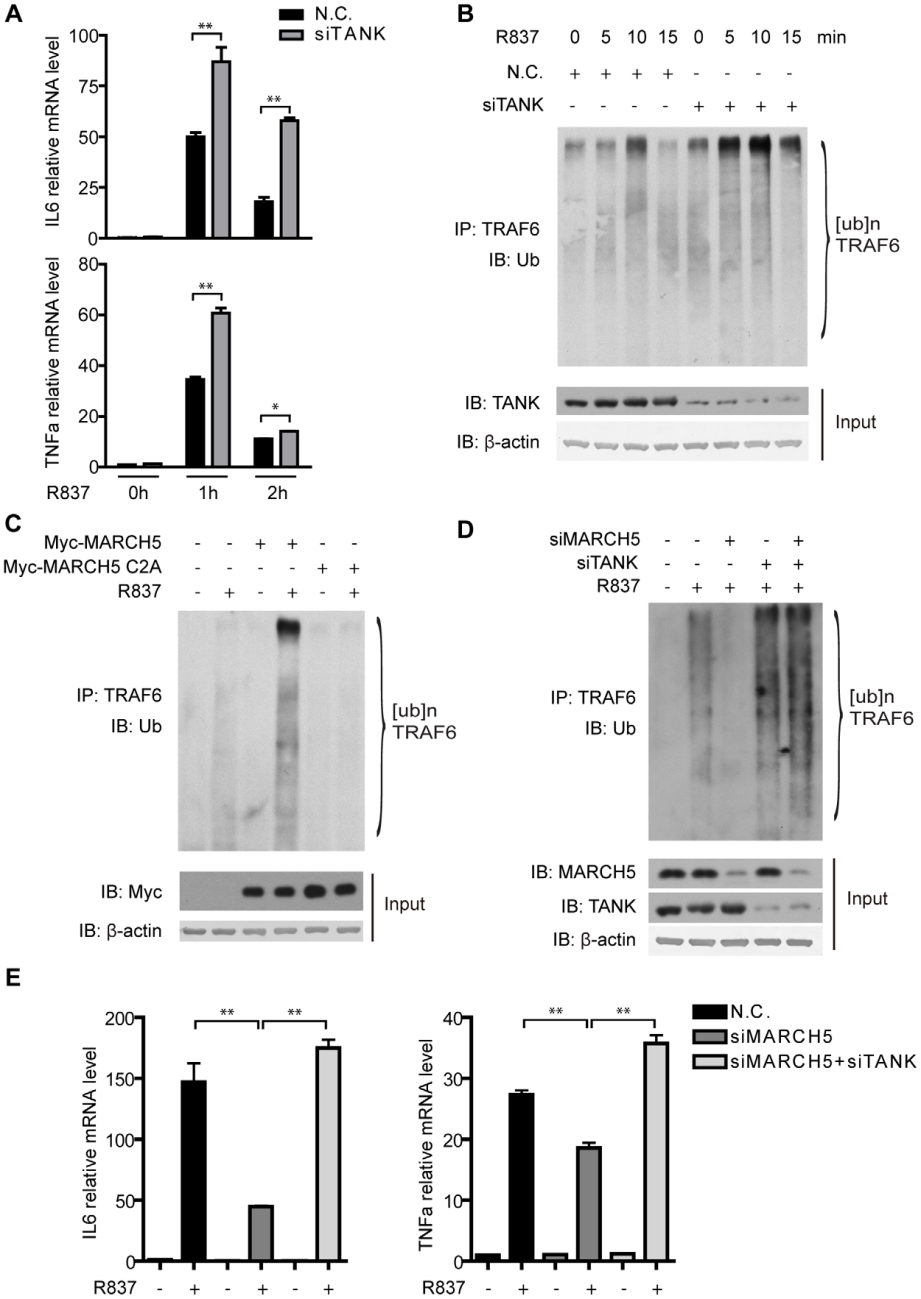
MARCH5 modulates the auto-ubiquitination of TRAF6. A, Raw264.7 cells were transfected with the negative control (N.C.) or TANK siRNA. Then the cells were treated with R837 (10 µg/ml) for the indicated time periods. Induction of IL6 and TNFα mRNA was measured by quantitative PCR. B, Raw264.7 cells were transfected with the indicated siRNAs and then stimulated with R837 (10 µg/ml) for the indicated time periods. Cell lysates were subjected to immunoprecipitation and then immunoblotted with the indicated antibodies. C, Raw264.7 cells were transfected with the empty vector or the indicated plasmids and then stimulated with R837 (10 µg/ml) for 5 minutes. Cell lysates were subjected to immunoprecipitation and then immunoblotted with the indicated antibodies. D, Raw264.7 cells were transfected with the indicated siRNAs and then stimulated with R837 (10 µg/ml) for 7.5 minutes. Cell lysates were subjected to immunoprecipitation and then immunoblotted with the indicated antibodies. E, The indicated siRNAs were transfected into Raw264.7 cells. Induction of IL6 and TNFα mRNA was measured by quantitative PCR after R837 (10 µg/ml) stimulation. Data from A and E are presented as means ± S.D. from three independent experiments. *, *P*<0.05; **, *P*<0.01.

Recently, TANK was found to negatively regulate TLR-mediated induction of pro-inflammatory cytokines [19]. We confirmed this observation in Raw264.7 cells, i.e., knockdown of TANK resulted in augmented cytokine production (IL6 and TNFα) in response to R837 stimulation (Fig. 6A). As TRAF6 is auto-ubiquitinated in response to TLR stimuli, we tested whether TANK influenced the auto-ubiquitination of TRAF6. Interestingly, R837-induced TRAF6 auto-ubiquitination was enhanced upon endogenous TANK depletion (Fig. 6B). This indicated that TANK inhibited NF-κB activation by suppressing TRAF6 auto-ubiquitination.

We went on to address whether TANK ubiquitination could release its inhibitory effects toward TRAF6. Raw264.7 cells were transfected with control or MARCH5 plasmids followed by R837 stimulation. Intriguingly, TRAF6 auto-ubiquitination was enhanced in the presence of the wild type MARCH5 (Fig. 6C). In contrast, the ubiquitination of TRAF6 was not affected when MARCH5 C2A was introduced (Fig. 6C).

Reciprocally, knockdown of MARCH5 inhibited the ubiquitination of TRAF6 (Fig. 6D). Notably, suppression of TRAF6 ubiquitination caused by MARCH5 knockdown could be reversed by simultaneously knocking down TANK, suggesting that MARCH5 regulated the auto-ubiquitination of TRAF6 through TANK. This was further supported by probing TLR7-mediated gene expression (IL6 and TNFα) when knocking down TANK and MARCH5 at the same time (Fig. 6E). Taken together, these data indicate that MARCH5 potentiates TLR7 signaling by releasing the inhibitory effects of TANK toward TRAF6.

### Mislocalization of MARCH5 impairs its function

**Figure 7:**
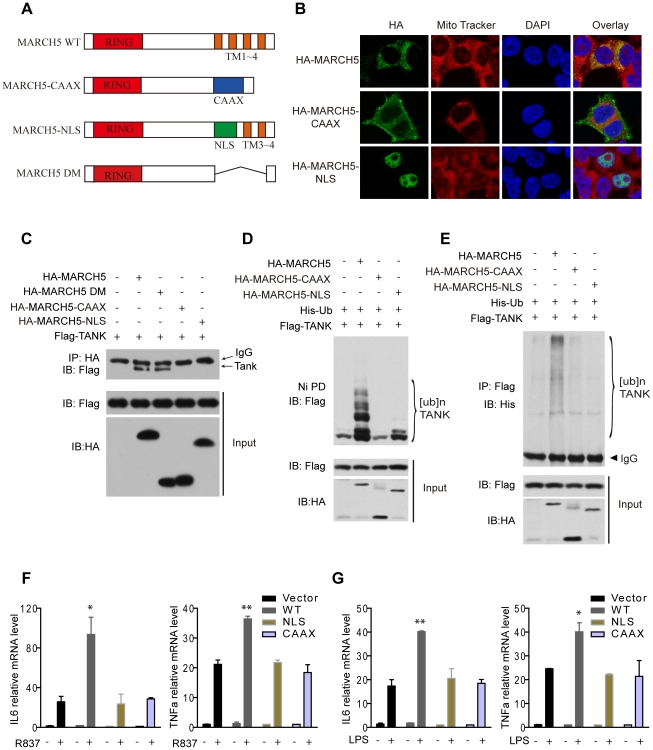
Mislocalization of MARCH5 impairs its function. A, Schematic diagram of wild-type MARCH5 (MARCH5 WT), its mislocalization mutants and the C-terminal transmembrane domain truncation (Δ105-246 a.a.) (MARCH5-DM); (RING, RING finger domain; TM, transmembrane domain; NLS, nuclear localization sequence). B, HA-MARCH5, HA-MARCH5-CAAX and HA-MARCH5-NLS were transfected into HEK293T cells individually, which were then stained with an anti-HA antibody and imaged by confocal microscopy. The mitochondria were stained with MitoTracker. C, HEK293T cells were transfected with the indicated constructs. Then, equal amounts of cell lysates were immunoprecipitated with an anti-HA antibody. The immunoprecipitates were immunoblotted with an anti-Flag antibody. D and E, HEK293T cells were transfected with the indicated plasmids. Twenty-four hours after transfection, cell lysates were subjected to Ni-NTA pulldown (Ni PD) (D) or immunoprecipitation (E) and then immunoblotted with the indicated antibodies. F and G, Equal amounts of the indicated Myc-tagged MARCH5 constructs were transfected into Raw264.7 cells. Induction of IL6 and TNFα mRNA by R837 (10 µg/ml) (F) or LPS (100 ng/ml) (G) stimulation was measured by quantitative PCR. Data from F and G are presented as means ± S.D. from three independent experiments. *, *P*<0.05; **, *P*<0.01. WT, wild type MARCH5; CAAX, MARCH5-CAAX; NLS, MARCH5-NLS.

To determine the importance of mitochondrial localization for MARCH5 function, we generated two mislocalization mutants of MARCH5. MARCH5 CAAX was constructed by replacing its C-terminal transmembrane domain with a targeting sequence to the plasma membrane, and MARCH5 NLS was constructed by replacing the same transmembrane domain with a nuclear localization sequence (Fig. 7A). As expected, MARCH5 CAAX and MARCH5 NLS were targeted to the plasma membrane and nucleus, respectively (Fig. 7B middle and lower panel). Interestingly, MARCH5 CAAX and MARCH5 NLS failed to interact with TANK, whereas the MARCH5-DM, the C-terminal transmembrane domain truncation, could still bind TANK (Fig. 7C). Corroborating the activity of the mislocalization mutants, neither MARCH5 CAAX nor MARCH5 NLS could catalyze the poly-ubiquitination of TANK (Fig. 7D, E). Furthermore, neither MARCH5 CAAX nor MARCH5 NLS could potentiate the expression of IL6 or TNFα induced by R837 or LPS (Fig. 7F, G). Collectively, these data indicate that mitochondrial localization of MARCH5 is essential for its regulatory function in innate immunity.

## Discussion

A new paradigm has been established in the past decade, revealing how Toll-like receptors (TLRs) detect a wide range of pathogens and then initiate immediate host defenses. As a result, cytokines and chemokines are induced to mobilize immune cells for controlling and eliminating pathological infections. Given that the TLR signal transduction cascade is the first line of the host defense against pathogens, they are subjected to multiple layers of positive and negative regulations. Herein, we characterize the mitochondrial protein MARCH5 as an essential and positive modulator of TLR7 signaling.

In this study, several lines of evidence substantiate the novel function of MARCH5 in TLR7 signaling. First, exogenous expression of MARCH5 potentiated the induction of NF-κB responsive genes upon R837 stimulation, but not the induction of IRF3/7 responsive genes. Second, knockdown of MARCH5 unequivocally resulted in the reduction of NF-κB-mediated gene expression, and this attenuation was rescued by exogenously expressing a siRNA-resistant rMARCH5. Third, MARCH5 interacted with TANK, a negative regulator of TLR7 signaling. Interestingly, TANK could partially co-localize to mitochondria in response to TLR7 stimulation. This interaction was increased upon R837 challenge, suggesting that the interaction was transient and dynamic. Fourth, knockdown of TANK impaired the ability of MARCH5 to potentiate TLR7 signaling.

Previous *in vitro* studies suggested that TANK positively regulates TBK1 and IKKε-mediated production of type I interferon [36]. However, analysis of TANK−/− mice indicated that TANK is not essential for the induction of type I interferon downstream of RIG-I/MDA5 or TRIF [19]. Further analysis revealed that TANK is critical for the negative regulation of canonical NF-κB activation via suppression of TRAF6 auto-ubiquitination [19]. The underlying mechanism is still not clear.

Ubiquitination is an effective mechanism to regulate TLR signaling pathways. E3 ubiquitin ligases (Nrdp1, A20) and de-ubiquitinases (DUBA, CYLD, and A20) have been demonstrated as positive or negative modulators of these pathways. Nrdp1 ‘preferentially’ promotes TLR-mediated production of type I interferon [37]. A20 and CYLD ‘preferentially’ terminate TLR-induced activation of NF-κB by de-ubiquitinating their substrates, such as RIP1, TRAF6, TAK1, NEMO and so on [15], [16]. DUBA interacts with and de-ubiquitinates TRAF3, thereby attenuating TLR-dependent and TLR-independent antiviral responses [38].

K48-linked poly-ubiquitin chains usually target substrates for proteasome degradation, whereas K63-linked poly-ubiquitin chains usually regulate substrate activity but do not promote effective degradation. Gatot *et al.* previously reported that TANK is subjected to lipopolysaccharide mediated K63-linked poly-ubiquitination [35]. However, the identity of the relevant E3 ubiquitin ligase and the functional consequence of TANK poly-ubiquitination remained to be revealed. In this study, we showed that ectopic expression of MARCH5 enhanced poly-ubiquitination of TANK after R837 stimulation, whereas knockdown of MARCH5 attenuated this poly-ubiquitination. Wild type MARCH5 catalyzed the K63-linked poly-ubiquitination of TANK on its Lysines 229, 233, 280, 302 and 306. Neither MARCH5 C2A nor MARCH5 ΔRING could synergize the activation of NF-κB, stimulated by R837. The siRNA-resistant mutants (rMARCH5 C2A or rMARCH5 ΔRING) could not rescue the NF-κB activation in MARCH5 knockdown cells. In addition, TANK *per se* could interfere with TRAF6 auto-ubiquitination.

The *in vitro* ubiquitination assay revealed that MARCH5 could catalyze the formation of both K48- and K63-linked polyUb chains. This is somewhat unexpected given that MARCH5 mediates only K63-linked polyubiquitination of TANK in transfected cells. One possible explanation for this discrepancy is that additional cofactors may exist *in vivo* to guide the reaction of MARCH5 in favor of the K63 linkage. Indeed, we have previously shown that TRAF6 only catalyzes the formation of K63-linked polyUb chains *in vivo*, which positively regulate the NF-κB signaling pathway [39]. Similar to what is observed for MARCH5, TRAF6 could facilitate the assembly of both K48- and K63-linked polyUb chains *in vitro* [40]. Recent mechanistic studies of polyUb chain formation revealed that additional proteins were involved in the determination of polyUb linkage [41], [42]. Strikingly, the NEMO protein is reported to be modified *in vivo* by linear-, K27- and K63-linked polyUb chains under different physiological conditions [43]. It is thus a great challenge to monitor the dynamic formation of various polyUb chains of different linkages and determine their cognate functional consequences. Currently, we are trying to use the RNAi approach to screen for potential cofactor(s) of MARCH5. Hopefully, this will shed new light on how MARCH5 catalyzes the formation of K63-linked polyUb chains *in vivo*. Taken together, we propose that MARCH5 is an authentic E3 ubiquitin ligase and catalyzes K63-linked poly-ubiquitination of TANK. MARCH5 modulates TLR7 signaling via releasing the inhibitory action of TANK toward TRAF6.

We speculate that MARCH5 potentiates TLR7 and TLR4 signaling via a similar mechanism. Interestingly, this regulatory function was less potent for TLR4 signaling, probably due to the observation that other TRAFs could partially mediate TLR4 signaling to NF-κB activation [44]. Consistently, it appears that MARCH5 does not influence the activation of IRF3/7, since TANK displays no regulatory role toward TRAF3 [19].

Interestingly, mislocalization of MARCH5 to either the plasma membrane or the nucleus abolishes its function toward TLR7 signaling. An increasing number of sub-cellular organelles are functionally connected to the anti-microbial defense system. For example, it has been proposed that endosomes and lysosomes contain TLR3/7/8/9 and probably TLR4 [45]. Recognition of PAMPs by TLRs actually takes place inside these intracellular membrane structures, instead of on the plasma membrane. In addition, the endoplasmic reticulum (ER) plays an active role during the transport of TLRs to their appropriate locations. Unexpectedly, mitochondria have recently been uncovered as a new platform for sensing intracellular virus infections. Notably, the existence of cross-talk between TLR signaling and mitochondrial proteins remained unknown. Arguably, our current study reveals the first mitochondrial protein to positively regulate TLR signaling. Because mitochondria and the ER are physically connected, we expect that future investigations will uncover more intricate cross-talk between them during TLR signaling.

MARCH family proteins contain a RING finger domain and some trans-membrane motifs. Notably, many of the 11 mammalian MARCH proteins have been implicated in modulating immune functions, either directly or indirectly. MARCH8 (also known as c-MIR, cellular modifier of immune response) was demonstrated to specifically catalyze B7.2 ubiquitination and its subsequent lysosomal degradation. In addition, both MARCH8 and MARCH1 negatively modulate the expression of CD95 (Fas), TfR (transferrin receptor) and MHC class II [28]. MARCH4 and MARCH9 influence antigen presentation by MHC class I molecules. Furthermore, ectopic-expression of MARCH9 leads to the down-regulation of surface ICAM1, a co-stimulatory molecule for T and B cells [28]. It will be intriguing to examine whether other MARCH family proteins play a critical role in immune regulation.

## Materials and Methods

### Plasmids

MARCH5, TANK, GIDE, TRAF6, TRAF3, NEMO, MAVS and RIG-I cDNAs were amplified by PCR from thymus cDNA library (Clontech) and subsequently cloned into mammalian expression vectors as indicated. His-Ub and the reporter plasmids (κB-luciferase and PRDIII-I-luciferase reporters) have been described previously [46]. All point mutations were introduced by using a QuickChange XL site-directed mutagenesis method (Stratagene). All constructs were confirmed by sequencing.

### Reagents

Rabbit polyclonal anti-MARCH5 antibody was a gift from Shigehisa Hirose (Tokyo Institute of Technology, Midori-ku, Yokohama, Japan). Anti-TANK antibodies were raised in rabbits against full-length mouse TANK and affinity purified using an antigen column. Other commercially available antibodies and reagents used were as follows: HA, Myc, Tom20 and Ub antibodies were purchased from Santa Cruz Biotechnology, Inc. Tubulin, Flag and β-actin antibodies were obtained from Sigma-Aldrich. R837 and lipopolysaccharide were purchased from Sigma-Aldrich.

### Cell culture and transfection

HEK293T and RAW264.7 cells were cultured using DMEM (Invitrogen) plus 10% FBS (Hyclone), supplemented with 1% penicillin-streptomycin (Invitrogen). The procedure for generating BMDCs (bone marrow-derived dendritic cells) has been described previously [26]. Lipofectamine (Invitrogen) was used for transient transfection of HEK293T Cells. RAW264.7 cells were transfected with Nucleofector (Amaxa). Small interference RNA was transfected with Lipofectamine 2000 (Invitrogen) according to the manufacturer's instructions.

### Immuno-precipitation analysis and Immuno-blot analysis

For immuno-precipitation analysis, cells were lysed in TBS buffer (50 mM Tris-Cl pH 7.4, 150 mM NaCl) supplemented with 1% Triton-X 100, 1 mM PMSF and a protease inhibitor cocktail (Roche). After pre-clearing for 1 hour, lysates were incubated with the appropriate antibody for four hours to overnight at 4°C. Two hours after adding protein A/G agarose, the immuno-precipitates were extensively washed with lysis buffer and eluted with SDS loading buffer by boiling for 3 min.

For immuno-blot analysis, the samples were resolved by SDS-PAGE and transferred to a PVDF membrane (Millipore). Immunoblotting was probed with indicated antibodies. The proteins were visualized by using a NBT/BCIP Western blotting system (Promega) or a SuperSignal West Pico chemiluminescence ECL kit (Pierce).

### Ni-NTA pulldown analysis

For Ni-NTA pulldown analysis, cells were washed with PBS and then lysed in His-Lysis Buffer (50 mM Tris-Cl. pH 7.4, 6M Urea). 20 µL Ni-NTA agarose beads (Qiagen) were then added into the post-centrifuged lysates and rotated for four hours at 4°C. After extensively washing with His-Lysis Buffer, the precipitates were boiled with SDS loading buffer and then subjected to SDS-PAGE followed by immuno-blot analysis.

### Reporter assays

Cells were seeded in 12-well plates and transfected with reporter gene plasmids combined with siRNAs and other constructs as indicated. The total amount of DNA was kept constant by supplementing with empty vectors. pTK-Renilla was cotransfected to normalize transfection efficiency. Luciferase activity was analyzed with the Dual Luciferase Reporter Assay System (Promega).

### Real-time RT-PCR

Total RNA was extracted using TRIzol reagent (Invitrogen) according to the manufacturer's instruction. Reverse transcription of purified RNA was performed using oligonucleotide dT primer. The quantifications of gene transcripts were performed by real-time PCR using Power SYBR GREEN PCR MASTER MIX (ABI). All values were normalized to the level of β-actin mRNA. The primers used are as follows:

β-actin sense (5-AAAGACCTCTATGCCAACAC-3),
β-actin antisense (5- TCGTACTCCTGCTTGCTGAT-3);
IL6 sense (5- GAGAGGAGACTTCACAGAGGATAC-3),
IL6 antisense (5- GTACTCCAGAAGACCAGAGG-3);
TNFα sense (5- GACACCATGAGCACAGAAAG-3),
TNFα antisense (5- GAGTAGACAAGGTACAACCC-3);
ISG15 sense(5- GACACCATGAGCACAGAAAG-3),
ISG15 antisense (5- GAGTAGACAAGGTACAACCC-3).

### RNA interference

Chemically synthesized 21-nucleotide siRNA duplexes were obtained from Gene-Pharma and transfected using Lipofectamine 2000 (Invitrogen) according to the manufacturer's instructions. RNA oligonucleotides used in this study are as follows:

NC: 5-UUCUCCGAAGGUGUCACGU-3;
si-MARCH5 369#: 5-GGUUGUAGGCCAUAAAGAATT-3;
si-MARCH5 582#: 5-AGCUGAAGCUAACCCUUUATT-3;
si-hMARCH5: 5-GGGUGGAAUUGCGUUUGUUTT-3;
si-TANK: 5-GACUUUCUGGGACCUUAAATT-3.

### Confocal microscopy

Cells were fixed with 4% paraformaldehyde, permeabilized with 0.1% Triton X-100, blocked with 1% bovine serum albumin and stained with the indicated primary antibodies followed by FITC-conjugated secondary antibody (Jackson Immuno-Research Laboratories). Nuclei were counterstained with DAPI (Sigma-Aldrich). For mitochondria staining, living cells were incubated with 300 nM Mito Tracker Red (Invitrogen) for 30 min at 37°C. Slides were mounted by Aqua-Poly/Mount (Polysciences). Imaging of the cells was carried out using Leica laser scanning confocal microscopy.

### Cell cycle Analysis

Preparations of cell suspensions for cell cycle analysis were performed as previously described [47]. Briefly, cells were trypsinized and fixed in ice-cold 70% ethanol at 4°C. After being washed with PBS twice, DNA was stained with 20 µg/mL propidium iodide (Sigma-Aldrich) in the presence of 200 µg/mL RNase A (Fermentas). Data were acquired on a FACSCalibur (BD Biosciences) and analyzed using CellQuest (BD Biosciences) and FlowJo (TreeStar Inc.).

### *In vitro* ubiquitination assay

For the synthesis of polyUb chains, purified MBP-MARCH5-NT (0.1 µM) was mixed with E1 (50 nM), E2 (0.3 µM), ubiquitin or ubiquitin mutants (0.1 mM) (Boston Biochem) in a reaction buffer containing 50 mM Tris–HCl, pH 7.5, 5 mM MgCl_2_, 2 mM ATP, 2 mM DTT. The reaction was carried out at 30°C for 90 min and then resolved by SDS-PAGE. Ubiquitinated products were detected by immunoblotting with a Ub-specific antibody (P4D1).

### Measurement of cytokines

Concentrations of cytokines in culture supernatants were measured by ELISA kits (R&D Systems) according to the manufacturer's instructions.

### Sub-cellular fractionation

HEK293T cells were washed with cold PBS and lysed by douncing in homogenization buffer (buffer H: 210 mM sucrose, 70 mM mannitol, 1 mM EDTA, 1 mM EGTA, 1.5 mM MgCl_2_, 10 mM Hepes (pH 7.2), protease inhibitor cocktail). The homogenate was centrifuged at 500×g for 10 min, and the pellet (P1) was saved as crude nuclei. The supernatant (S1) was centrifuged at 5,000×g for 10 min to precipitate mitochondria (P5). The whole cell and fractions of P5 and S5 were lysed in lysis buffer (50 mM Tris-HCl. pH 7.4, 150 mM NaCl, 1 mM EDTA, and 1% Triton-X 100, protease inhibitor cocktail) followed by immunoblot analysis.

### Statistics

Student's t test was used for the statistical analysis of two independent treatments. For all tests, a P value of <0.05 was considered statistically significant.

### Accession numbers

The GenBank (http://www.ncbi.nlm.nih.gov/Genbank) accession numbers for the genes and gene products discussed in this paper are:

MARCH5 (NM_027314, NP_081590), TANK (NM_001164071.1, NP_001157543.1), GIDE (NM_026689.3, NP_080965.2), TRAF6 (NM_004620.2, NP_004611.1), TRAF3 (NM_145725.2, NP_663777.1), NEMO (NM_001099857.1, NP_001093327.1), MAVS (NM_020746.4, NP_065797.2), RIG-I (NM_014314.3, NP_055129.2), TRIF (NM_182919.2, NP_891549.1), MyD88 (NM_002468.4, NP_002459.2), IRAK1 (NM_001025242.1, NP_001020413.1).

## Supporting Information

Figure S1 MARCH5 potentiates the expression of pro-inflammatory cytokines. A and B, Raw264.7 cells were transfected with the indicated plasmids. After R837 (10 µg/ml) (A) or LPS (100 ng/ml) (B) stimulation, IL6 and TNFα production was determined by ELISA. C and D, Raw264.7 cells were transfected with the indicated siRNAs. TNFα and IL-6 production was measured by ELISA after R837 (10 µg/ml) (C) or LPS (100 ng/ml) (D) treatment. Data from A–D are presented as means ± S.D. from three independent experiments. *, *P*<0.05; **, *P*<0.01. N.D., not detected.
(TIF)

Figure S2 Ectopic expression of MARCH5 displays no effect on cell cycling. A and B, Representative DNA histogram of PI fluorescence in cells, as assessed by FACS. Equal amounts of the indicated constructs were transfected into Raw264.7 cells. Twenty-four hours after transfection, cells were stimulated with R837 (10 µg/ml) (A) or LPS (100 ng/ml) (B) before cell cycle analysis was performed. C, the ratio of cells in G0–G1 phase, S phase and G2-M phase of the cell cycle was measured by FACS and analyzed by FlowJo software. Data are presented as means ± S.D. from three independent experiments.
(TIF)

Figure S3 Knockdown of MARCH5 does not affect cell cycling. A and B, Representative DNA histogram of PI fluorescence in cells, as assessed by FACS. Raw264.7 cells were transfected with the indicated siRNAs. Cell cycle analysis was performed after R837 (10 µg/ml) (A) or LPS (100 ng/ml) (B) stimulation. C, Ratio of cells in G0–G1 phase, S phase and G2-M phase of the cell cycle was measured by FACS and analyzed by FlowJo software. Data are presented as means ± S.D. from three independent experiments.
(TIF)

Figure S4 TANK is partially co-localized with mitochondria upon TLR7 stimulation. A and B, After stimulation by R837 or LPS, RAW264.7 cells were immunostained with anti-TANK (A) or anti-TRAF6 (B) antibodies as indicated, and then imaged by confocal microscopy. The mitochondria were stained with MitoTracker. Arrows indicate representative co-localization between TANK and mitochondria.
(TIF)

Figure S5 MARCH5 facilitates the assembly of both K48- and K63-Linked polyUb Chains *in vitro*. A, Synthesis of polyUb chains by MARCH5-NT (MARCH5 1-96a.a.) but not MARCH5-NT C2A or MARCH5-NT ΔRING. The ubiquitination reaction contains E1, E2, Ub, and MARCH5-NT or MARCH5 RING-finger mutants (MARCH5-NT C2A and MARCH5-NT ΔRING) as indicated. PolyUb chains were detected by immunoblotting with a Ub-specific antibody. B and C, MARCH5-NT catalyzed the formation of both K63- (B) and K48-linked (C) polyUb chains. K48 and K63: all lysines on Ub are mutated to arginine except for K48 or K63.
(TIF)

Figure S6 TANK(5M) mutant could interact with MARCH5. HEK293T cells were cotransfected with the indicated constructs. Then, equal amounts of cell lysates were immunoprecipitated with an anti-Flag antibody. The immunoprecipitates were immunoblotted with an anti-HA antibody.
(TIF)

Figure S7 MARCH5 does not catalyze the poly-ubiquitination of RIG-I, MAVS, or TRAF3. HEK293T cells were transfected with the indicated plasmids. Twenty-four hours after transfection, cell lysates were subjected to immunoprecipitation and then immunoblotted with the indicated antibodies.
(TIF)

## References

[1] Akira S, Uematsu S, Takeuchi O (2006). Pathogen recognition and innate immunity.. Cell.

[2] Kawai T, Akira S (2010). The role of pattern-recognition receptors in innate immunity: update on Toll-like receptors.. Nat Immunol.

[3] Medzhitov R (2007). Recognition of microorganisms and activation of the immune response.. Nature.

[4] Diebold SS, Kaisho T, Hemmi H, Akira S, Reis e Sousa C (2004). Innate antiviral responses by means of TLR7-mediated recognition of single-stranded RNA.. Science.

[5] Heil F, Hemmi H, Hochrein H, Ampenberger F, Kirschning C (2004). Species-specific recognition of single-stranded RNA via toll-like receptor 7 and 8.. Science.

[6] Yang K, Puel A, Zhang S, Eidenschenk C, Ku CL (2005). Human TLR-7-, -8-, and -9-mediated induction of IFN-alpha/beta and -lambda Is IRAK-4 dependent and redundant for protective immunity to viruses.. Immunity.

[7] Kawai T, Sato S, Ishii KJ, Coban C, Hemmi H (2004). Interferon-alpha induction through Toll-like receptors involves a direct interaction of IRF7 with MyD88 and TRAF6.. Nat Immunol.

[8] Hoshino K, Sugiyama T, Matsumoto M, Tanaka T, Saito M (2006). IkappaB kinase-alpha is critical for interferon-alpha production induced by Toll-like receptors 7 and 9.. Nature.

[9] Wang C, Deng L, Hong M, Akkaraju GR, Inoue J (2001). TAK1 is a ubiquitin-dependent kinase of MKK and IKK.. Nature.

[10] Kobayashi K, Hernandez LD, Galan JE, Janeway CA, Medzhitov R (2002). IRAK-M is a negative regulator of Toll-like receptor signaling.. Cell.

[11] Brint EK, Xu D, Liu H, Dunne A, McKenzie AN (2004). ST2 is an inhibitor of interleukin 1 receptor and Toll-like receptor 4 signaling and maintains endotoxin tolerance.. Nat Immunol.

[12] Wald D, Qin J, Zhao Z, Qian Y, Naramura M (2003). SIGIRR, a negative regulator of Toll-like receptor-interleukin 1 receptor signaling.. Nat Immunol.

[13] Nakagawa R, Naka T, Tsutsui H, Fujimoto M, Kimura A (2002). SOCS-1 participates in negative regulation of LPS responses.. Immunity.

[14] Lee EG, Boone DL, Chai S, Libby SL, Chien M (2000). Failure to regulate TNF-induced NF-kappaB and cell death responses in A20-deficient mice.. Science.

[15] Wertz IE, O'Rourke KM, Zhou H, Eby M, Aravind L (2004). De-ubiquitination and ubiquitin ligase domains of A20 downregulate NF-kappaB signalling.. Nature.

[16] Wilkinson KD (2003). Signal transduction: aspirin, ubiquitin and cancer.. Nature.

[17] Rothe M, Xiong J, Shu HB, Williamson K, Goddard A (1996). I-TRAF is a novel TRAF-interacting protein that regulates TRAF-mediated signal transduction.. Proc Natl Acad Sci U S A.

[18] Cheng G, Baltimore D (1996). TANK, a co-inducer with TRAF2 of TNF- and CD 40L-mediated NF-kappaB activation.. Genes Dev.

[19] Kawagoe T, Takeuchi O, Takabatake Y, Kato H, Isaka Y (2009). TANK is a negative regulator of Toll-like receptor signaling and is critical for the prevention of autoimmune nephritis.. Nat Immunol.

[20] Scott I (2009). Mitochondrial factors in the regulation of innate immunity.. Microbes Infect.

[21] Zhong B, Yang Y, Li S, Wang YY, Li Y (2008). The adaptor protein MITA links virus-sensing receptors to IRF3 transcription factor activation.. Immunity.

[22] Onoguchi K, Onomoto K, Takamatsu S, Jogi M, Takemura A (2010). Virus-infection or 5'ppp-RNA activates antiviral signal through redistribution of IPS-1 mediated by MFN1.. PLoS Pathog.

[23] Moore CB, Bergstralh DT, Duncan JA, Lei Y, Morrison TE (2008). NLRX1 is a regulator of mitochondrial antiviral immunity.. Nature.

[24] Ishikawa H, Barber GN (2008). STING is an endoplasmic reticulum adaptor that facilitates innate immune signalling.. Nature.

[25] Lin R, Paz S, Hiscott J (2010). Tom70 imports antiviral immunity to the mitochondria.. Cell Res.

[26] Liu XY, Wei B, Shi HX, Shan YF, Wang C (2010). Tom70 mediates activation of interferon regulatory factor 3 on mitochondria.. Cell Res.

[27] Yang K, Shi HX, Qi R, Sun SG, Tang YJ (2006). Hsp90 regulates activation of interferon regulatory factor 3 and TBK-1 stabilization in Sendai virus-infected cells.. Molecular Biology of the Cell.

[28] Wang X, Herr RA, Hansen T (2008). Viral and cellular MARCH ubiquitin ligases and cancer.. Semin Cancer Biol.

[29] Karbowski M, Neutzner A, Youle RJ (2007). The mitochondrial E3 ubiquitin ligase MARCH5 is required for Drp1 dependent mitochondrial division.. J Cell Biol.

[30] Nakamura N, Kimura Y, Tokuda M, Honda S, Hirose S (2006). MARCH-V is a novel mitofusin 2- and Drp1-binding protein able to change mitochondrial morphology.. EMBO Rep.

[31] Yonashiro R, Ishido S, Kyo S, Fukuda T, Goto E (2006). A novel mitochondrial ubiquitin ligase plays a critical role in mitochondrial dynamics.. EMBO J.

[32] You F, Sun H, Zhou X, Sun W, Liang S (2009). PCBP2 mediates degradation of the adaptor MAVS via the HECT ubiquitin ligase AIP4.. Nat Immunol.

[33] Paz S, Vilasco M, Arguello M, Sun Q, Lacoste J (2009). Ubiquitin-regulated recruitment of IkappaB kinase epsilon to the MAVS interferon signaling adapter.. Mol Cell Biol.

[34] Arimoto K, Takahashi H, Hishiki T, Konishi H, Fujita T (2007). Negative regulation of the RIG-I signaling by the ubiquitin ligase RNF125.. Proc Natl Acad Sci U S A.

[35] Gatot JS, Gioia R, Chau TL, Patrascu F, Warnier M (2007). Lipopolysaccharide-mediated interferon regulatory factor activation involves TBK1-IKKepsilon-dependent Lys(63)-linked polyubiquitination and phosphorylation of TANK/I-TRAF.. J Biol Chem.

[36] Guo B, Cheng G (2007). Modulation of the interferon antiviral response by the TBK1/IKKi adaptor protein TANK.. J Biol Chem.

[37] Wang C, Chen T, Zhang J, Yang M, Li N (2009). The E3 ubiquitin ligase Nrdp1 ‘preferentially’ promotes TLR-mediated production of type I interferon.. Nat Immunol.

[38] Kayagaki N, Phung Q, Chan S, Chaudhari R, Quan C (2007). DUBA: a deubiquitinase that regulates type I interferon production.. Science.

[39] Deng L, Wang C, Spencer E, Yang L, Braun A (2000). Activation of the IkappaB kinase complex by TRAF6 requires a dimeric ubiquitin-conjugating enzyme complex and a unique polyubiquitin chain.. Cell.

[40] Yang K, Zhu J, Sun S, Tang Y, Zhang B (2004). The coiled-coil domain of TRAF6 is essential for its auto-ubiquitination.. Biochem Biophys Res Commun.

[41] Hochstrasser M (2006). Lingering mysteries of ubiquitin-chain assembly.. Cell.

[42] Koegl M, Hoppe T, Schlenker S, Ulrich HD, Mayer TU (1999). A novel ubiquitination factor, E4, is involved in multiubiquitin chain assembly.. Cell.

[43] Iwai K, Tokunaga F (2009). Linear polyubiquitination: a new regulator of NF-kappaB activation.. EMBO Rep.

[44] Hacker H, Redecke V, Blagoev B, Kratchmarova I, Hsu LC (2006). Specificity in Toll-like receptor signalling through distinct effector functions of TRAF3 and TRAF6.. Nature.

[45] Blasius AL, Beutler B (2010). Intracellular toll-like receptors.. Immunity.

[46] Shi HX, Yang K, Liu X, Liu XY, Wei B (2010). Positive regulation of interferon regulatory factor 3 activation by Herc5 via ISG15 modification.. Mol Cell Biol.

[47] Carey KD, Garton AJ, Romero MS, Kahler J, Thomson S (2006). Kinetic analysis of epidermal growth factor receptor somatic mutant proteins shows increased sensitivity to the epidermal growth factor receptor tyrosine kinase inhibitor, erlotinib.. Cancer Res.

