# Supplementary figures and images for: Mitochondrial Ubiquitin Ligase MARCH5 Promotes TLR7 Signaling by Attenuating TANK Action
Source: PLoS Pathog. 2011 May 19;7(5):e1002057. doi: 10.1371/journal.ppat.1002057 (PMC3098239; doi:10.1371/journal.ppat.1002057)

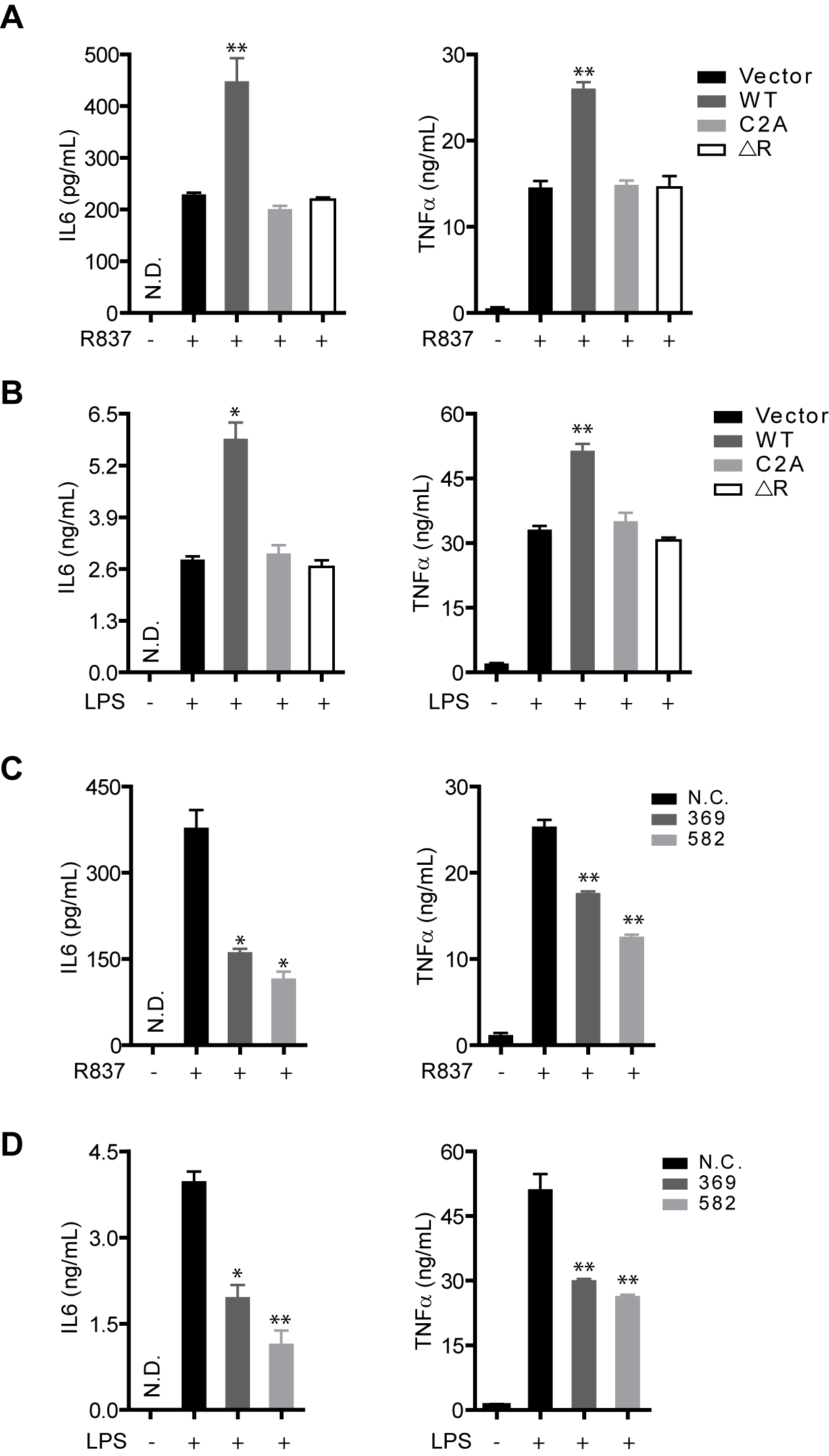

Supplement: Figure S1 — MARCH5 potentiates the expression of pro-inflammatory cytokines. A and B, Raw264.7 cells were transfected with the indicated plasmids. After R837 (10 µg/ml) (A) or LPS (100 ng/ml) (B) stimulation, IL6 and TNFα production was determined by ELISA. C and D, Raw264.7 cells were transfected with the indicated siRNAs. TNFα and IL-6 production was measured by ELISA after R837 (10 µg/ml) (C) or LPS (100 ng/ml) (D) treatment. Data from A–D are presented as means ± S.D. from three independent experiments. *, P<0.05; **, P<0.01. N.D., not detected. (TIF) [file ppat.1002057.s001.tif]

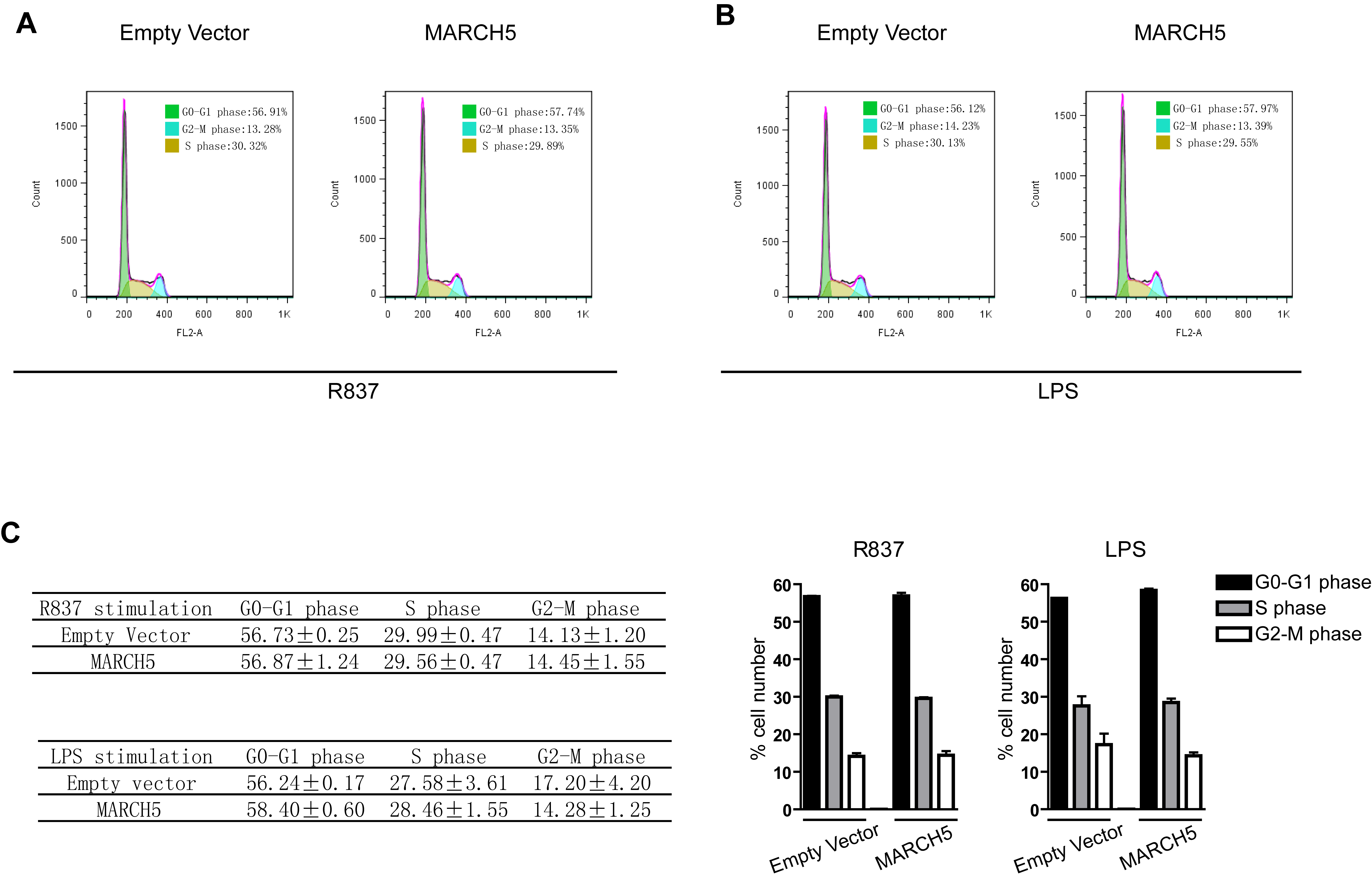

Supplement: Figure S2 — Ectopic expression of MARCH5 displays no effect on cell cycling. A and B, Representative DNA histogram of PI fluorescence in cells, as assessed by FACS. Equal amounts of the indicated constructs were transfected into Raw264.7 cells. Twenty-four hours after transfection, cells were stimulated with R837 (10 µg/ml) (A) or LPS (100 ng/ml) (B) before cell cycle analysis was performed. C, the ratio of cells in G0–G1 phase, S phase and G2-M phase of the cell cycle was measured by FACS and analyzed by FlowJo software. Data are presented as means ± S.D. from three independent experiments. (TIF) [file ppat.1002057.s002.tif]

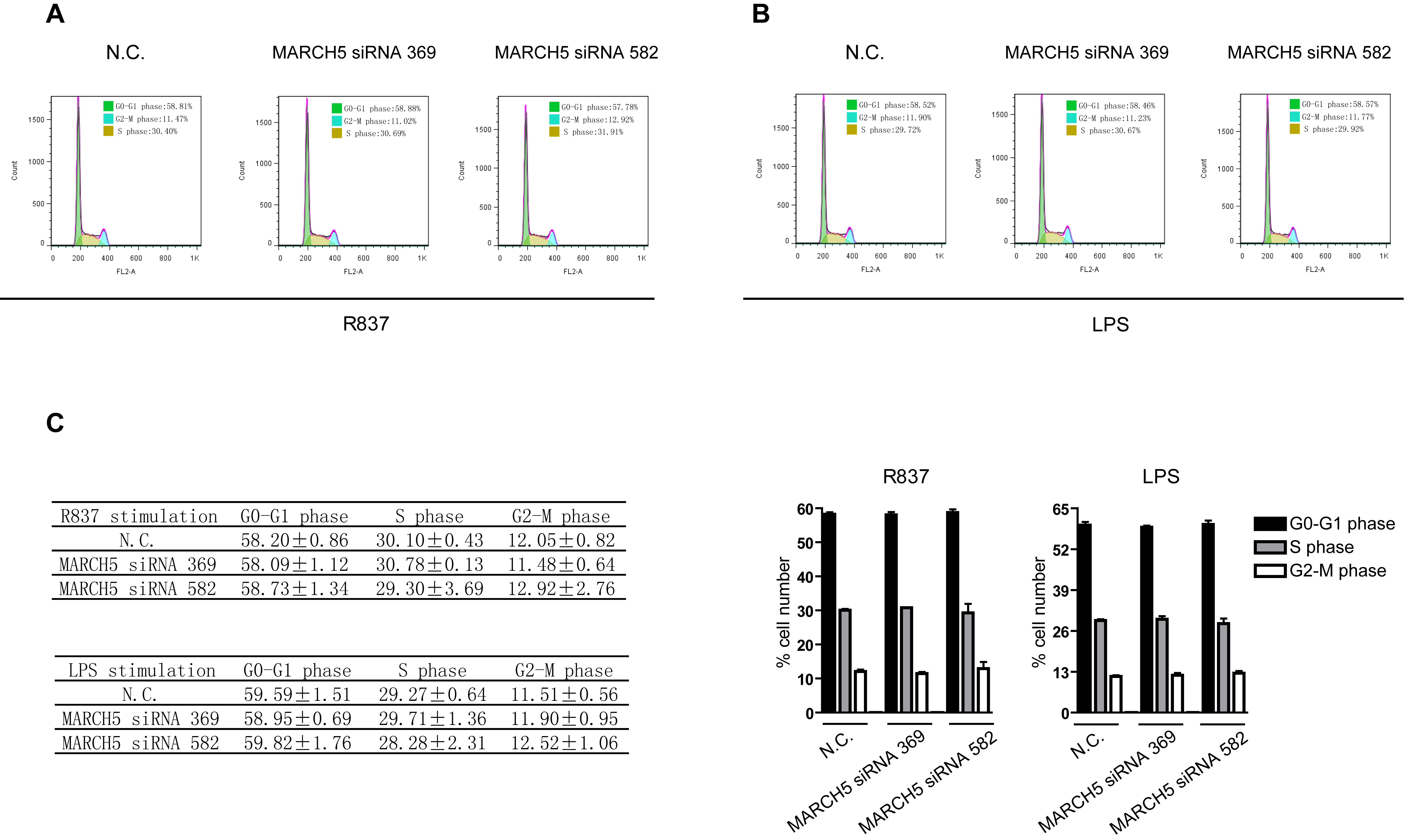

Supplement: Figure S3 — Knockdown of MARCH5 does not affect cell cycling. A and B, Representative DNA histogram of PI fluorescence in cells, as assessed by FACS. Raw264.7 cells were transfected with the indicated siRNAs. Cell cycle analysis was performed after R837 (10 µg/ml) (A) or LPS (100 ng/ml) (B) stimulation. C, Ratio of cells in G0–G1 phase, S phase and G2-M phase of the cell cycle was measured by FACS and analyzed by FlowJo software. Data are presented as means ± S.D. from three independent experiments. (TIF) [file ppat.1002057.s003.tif]

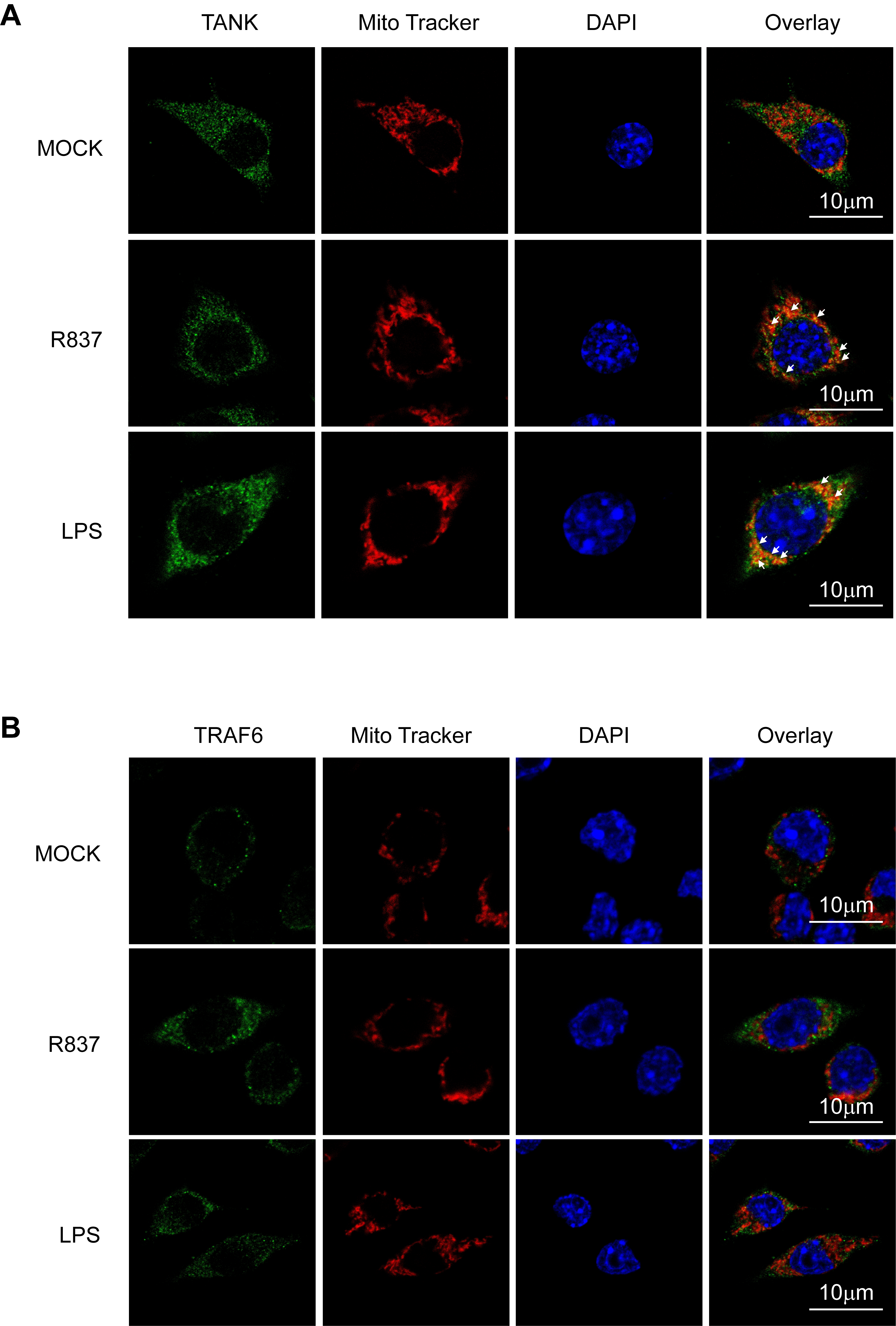

Supplement: Figure S4 — TANK is partially co-localized with mitochondria upon TLR7 stimulation. A and B, After stimulation by R837 or LPS, RAW264.7 cells were immunostained with anti-TANK (A) or anti-TRAF6 (B) antibodies as indicated, and then imaged by confocal microscopy. The mitochondria were stained with MitoTracker. Arrows indicate representative co-localization between TANK and mitochondria. (TIF) [file ppat.1002057.s004.tif]

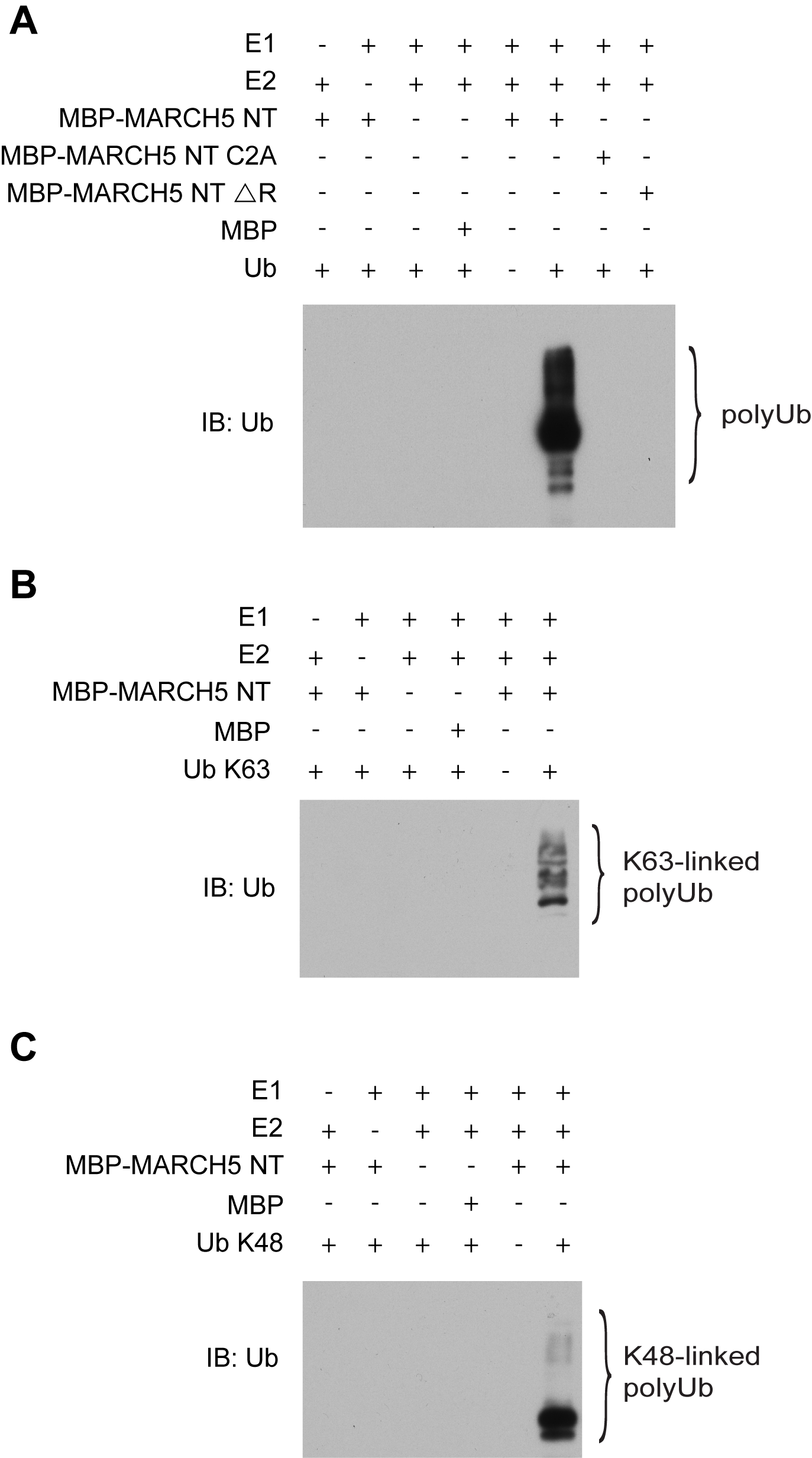

Supplement: Figure S5 — MARCH5 facilitates the assembly of both K48- and K63-Linked polyUb Chains in vitro. A, Synthesis of polyUb chains by MARCH5-NT (MARCH5 1-96a.a.) but not MARCH5-NT C2A or MARCH5-NT ΔRING. The ubiquitination reaction contains E1, E2, Ub, and MARCH5-NT or MARCH5 RING-finger mutants (MARCH5-NT C2A and MARCH5-NT ΔRING) as indicated. PolyUb chains were detected by immunoblotting with a Ub-specific antibody. B and C, MARCH5-NT catalyzed the formation of both K63- (B) and K48-linked (C) polyUb chains. K48 and K63: all lysines on Ub are mutated to arginine except for K48 or K63. (TIF) [file ppat.1002057.s005.tif]

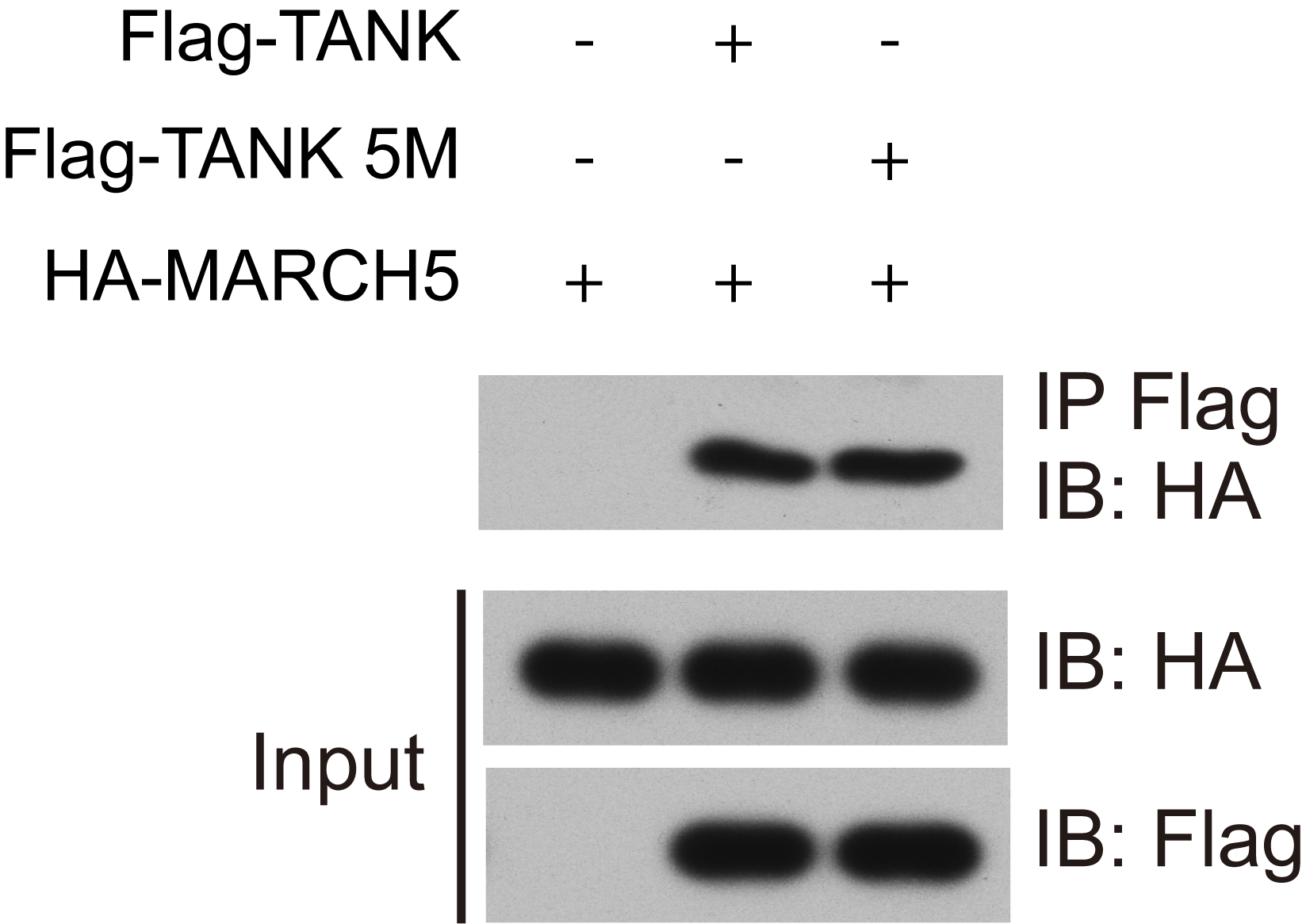

Supplement: Figure S6 — TANK(5M) mutant could interact with MARCH5. HEK293T cells were cotransfected with the indicated constructs. Then, equal amounts of cell lysates were immunoprecipitated with an anti-Flag antibody. The immunoprecipitates were immunoblotted with an anti-HA antibody. (TIF) [file ppat.1002057.s006.tif]

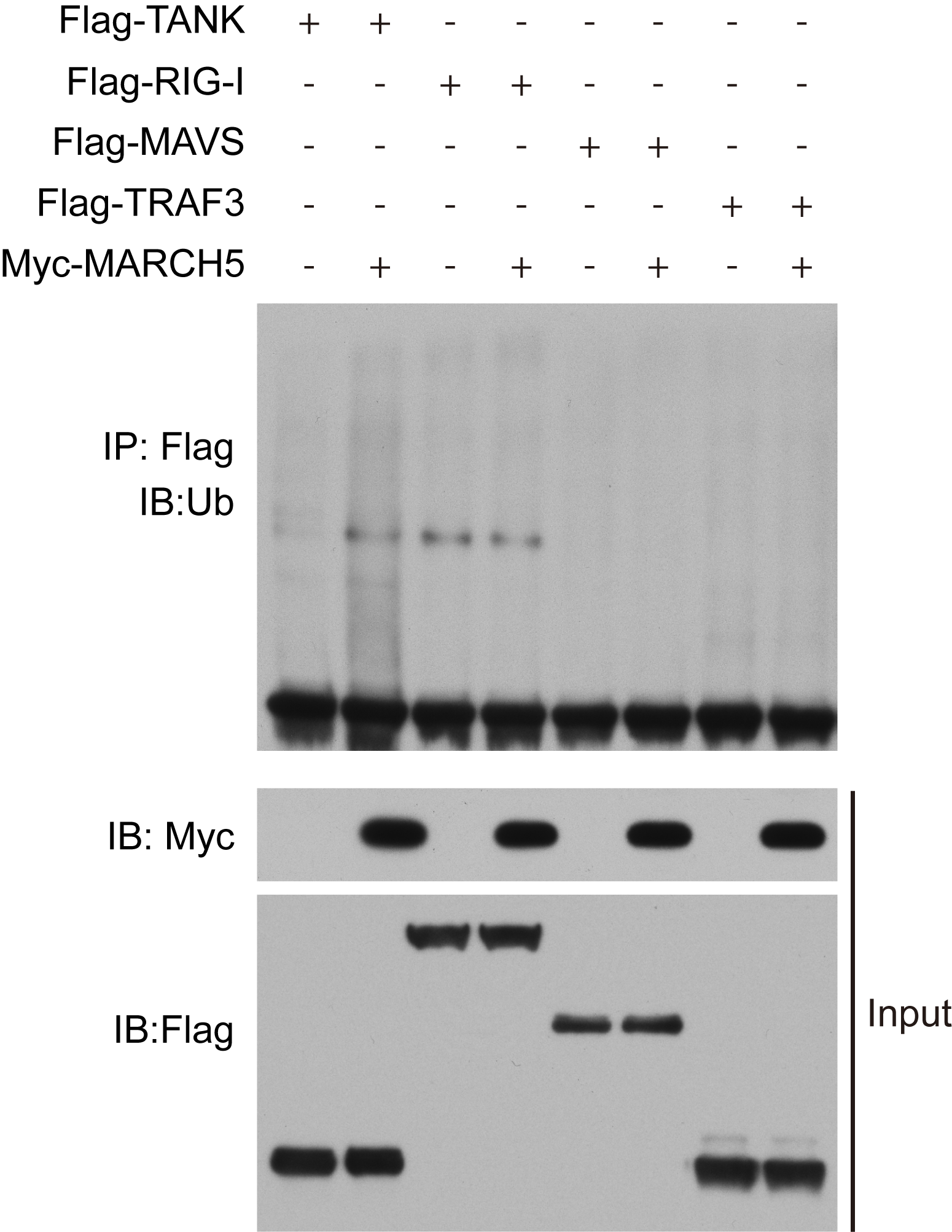

Supplement: Figure S7 — MARCH5 does not catalyze the poly-ubiquitination of RIG-I, MAVS, or TRAF3. HEK293T cells were transfected with the indicated plasmids. Twenty-four hours after transfection, cell lysates were subjected to immunoprecipitation and then immunoblotted with the indicated antibodies. (TIF) [file ppat.1002057.s007.tif]
